# Supplementary material for: High-Content Imaging Platform for Profiling Intracellular Signaling Network Activity in Living Cells
Source: Cell Chem Biol. 2016 Dec 22;23(12):1550–9. doi: 10.1016/j.chembiol.2016.11.008 (PMC5193178; doi:10.1016/j.chembiol.2016.11.008)
Supplement: Document S1. Figures S1–S14 and Table S1 [file mmc1.pdf]

Cell Chemical Biology, Volume *23*

Supplemental Information

High-Content Imaging Platform for Profiling Intracellular Signaling Network Activity in Living Cells

Dmitry Kuchenov, Vibor Laketa, Frank Stein, Florian Salopiata, Ursula Klingmüller, and Carsten Schultz

Supplementary Figure 1

Image processing step: | **sample image:**

1. Open images

Nuclei | Donor | Acceptor

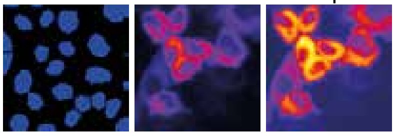

2. Background subtraction
a) “Triangle” threshold method
b) Subtract measured ‘Mean’ background

Nuclei | Donor | Acceptor

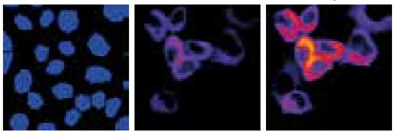

3. Noise reduction
“Median” filter
(radius size = 3)

Nuclei | Donor | Acceptor

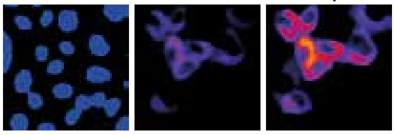

4. Thresholding
a) 32-bit conversion
b) Thresholding (”Huang” method)
c) Convert background to NaN

Donor | Acceptor

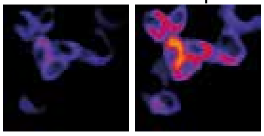

5. Calculating ratio image
(Donor channel/Acceptor channel)

Ratio

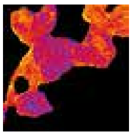

6. Cell segmentation
a) Z-projection of time points by average intencity
b) Thresholding (”Huang” method)
c) Watershed and Voronoi algorithms
d) Analyze Particles (Size: 70-1000, circularity: 0-0.8)

Nuclei | Donor | Acceptor

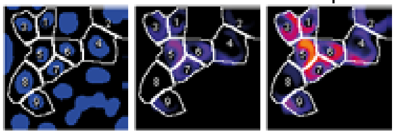

7. Parameter extraction

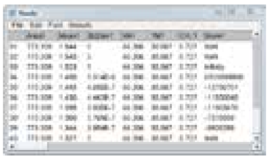

Supplementary Figure 2

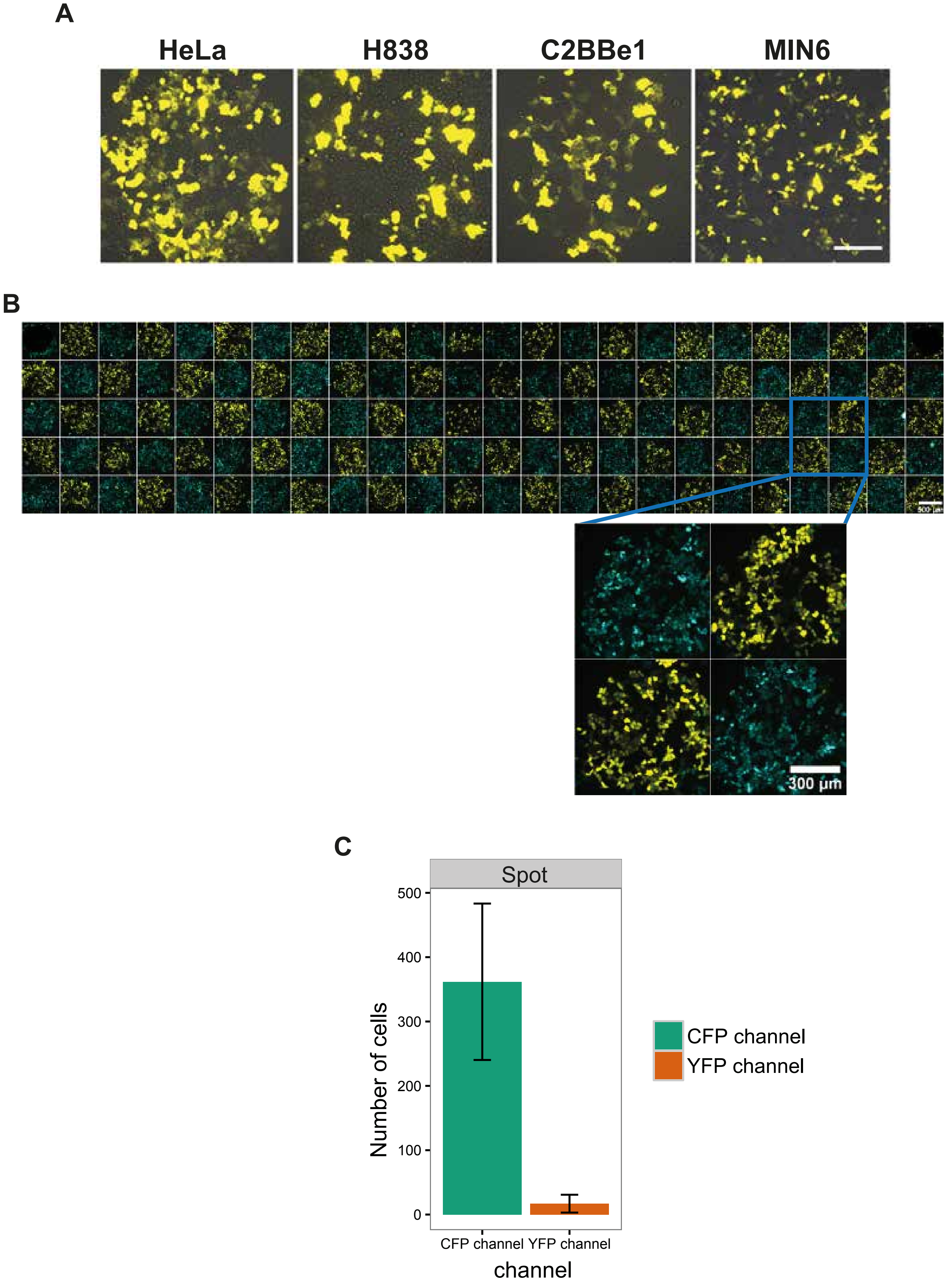

Supplementary Figure 3

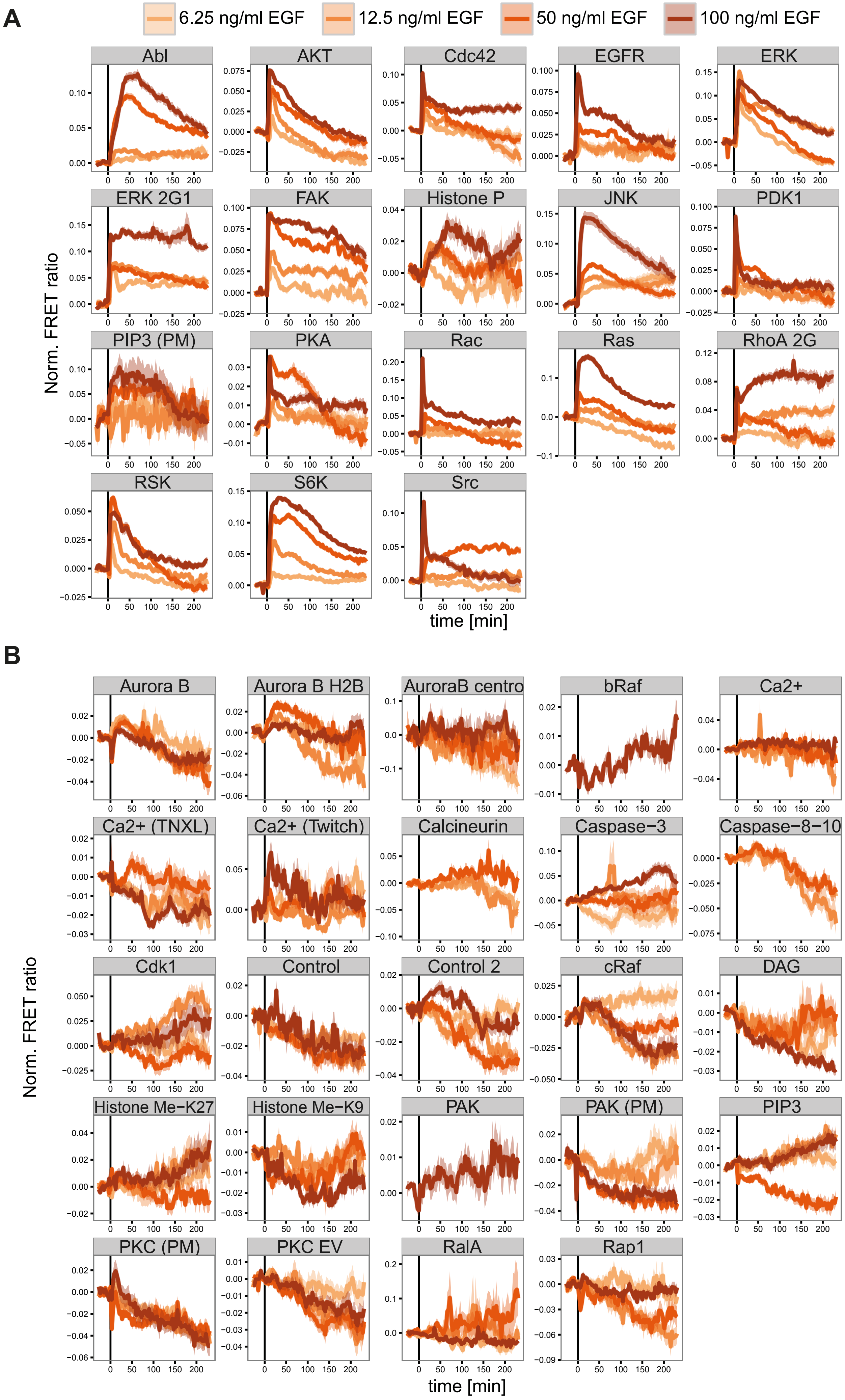

Supplementary Figure 4

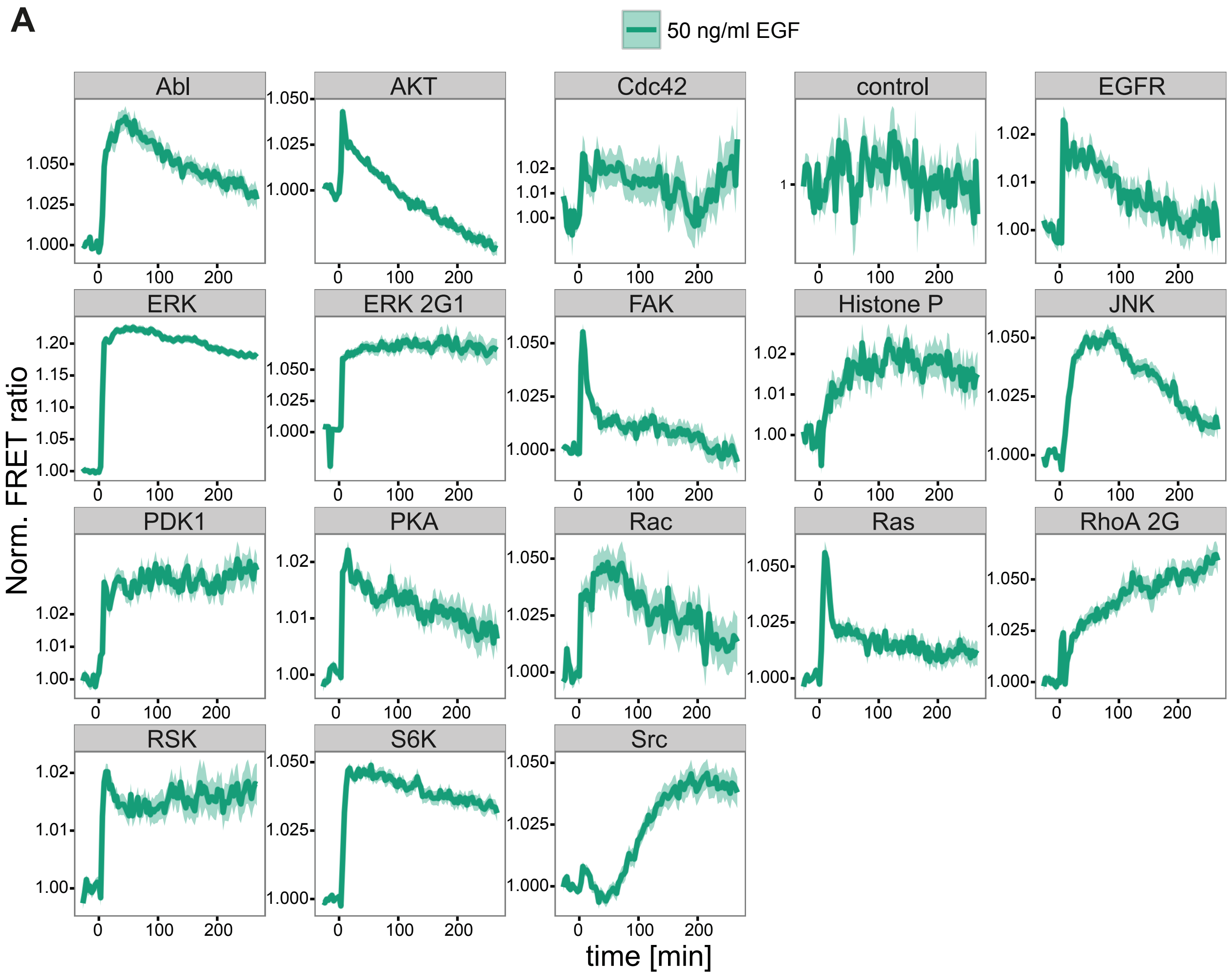

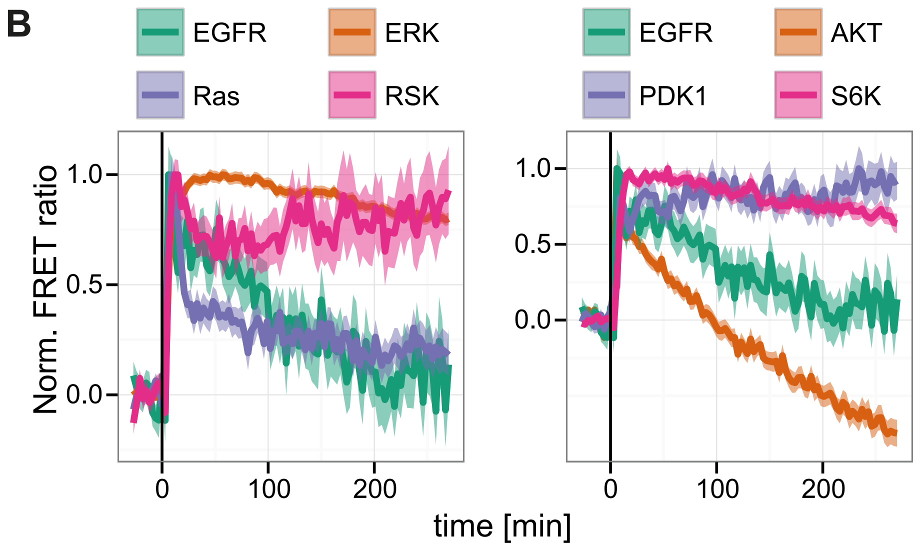

Supplementary Figure 5

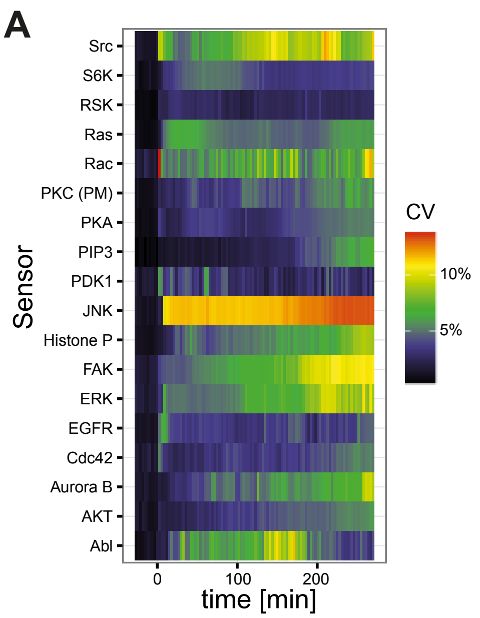

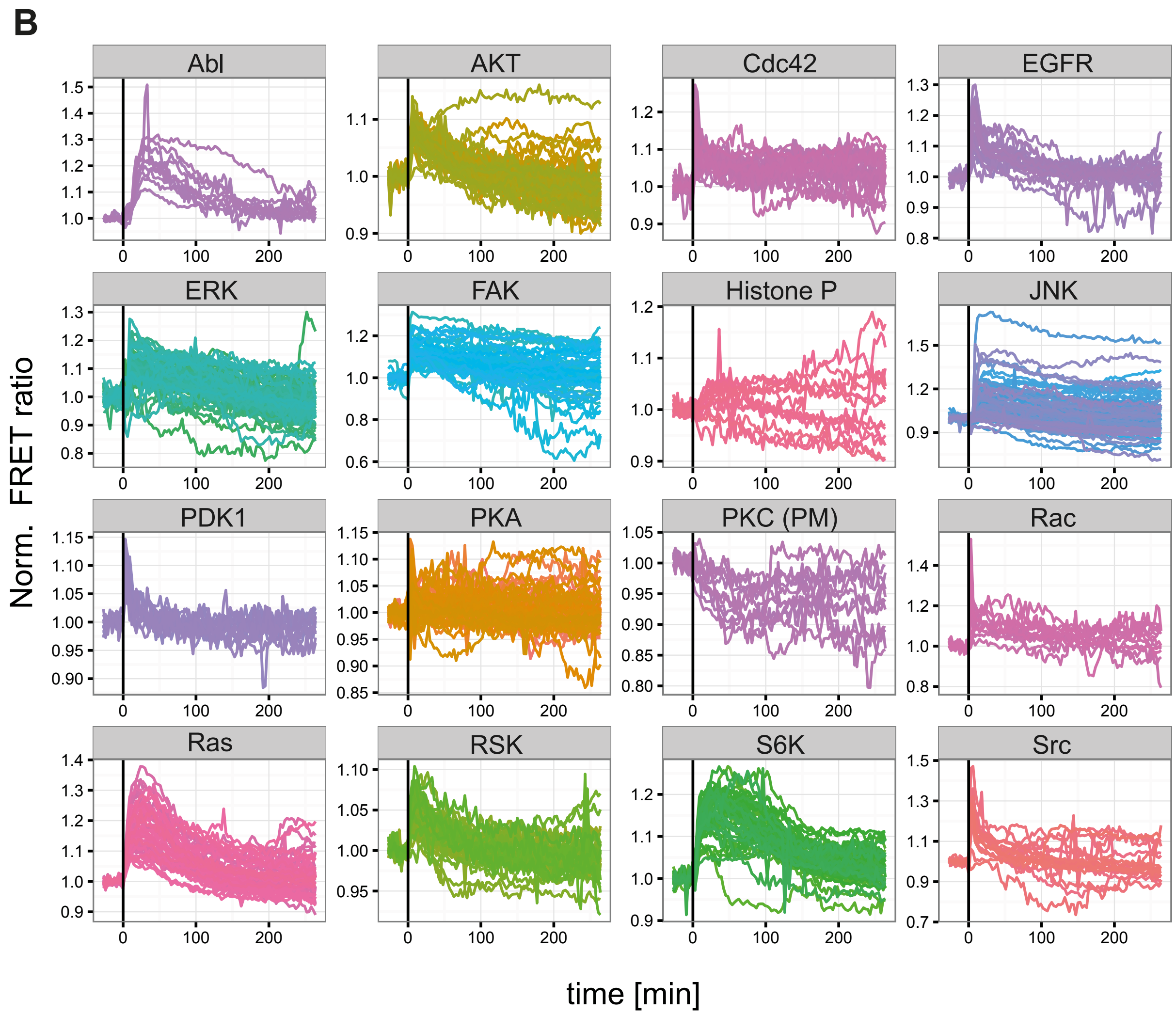

Supplementary Figure 6

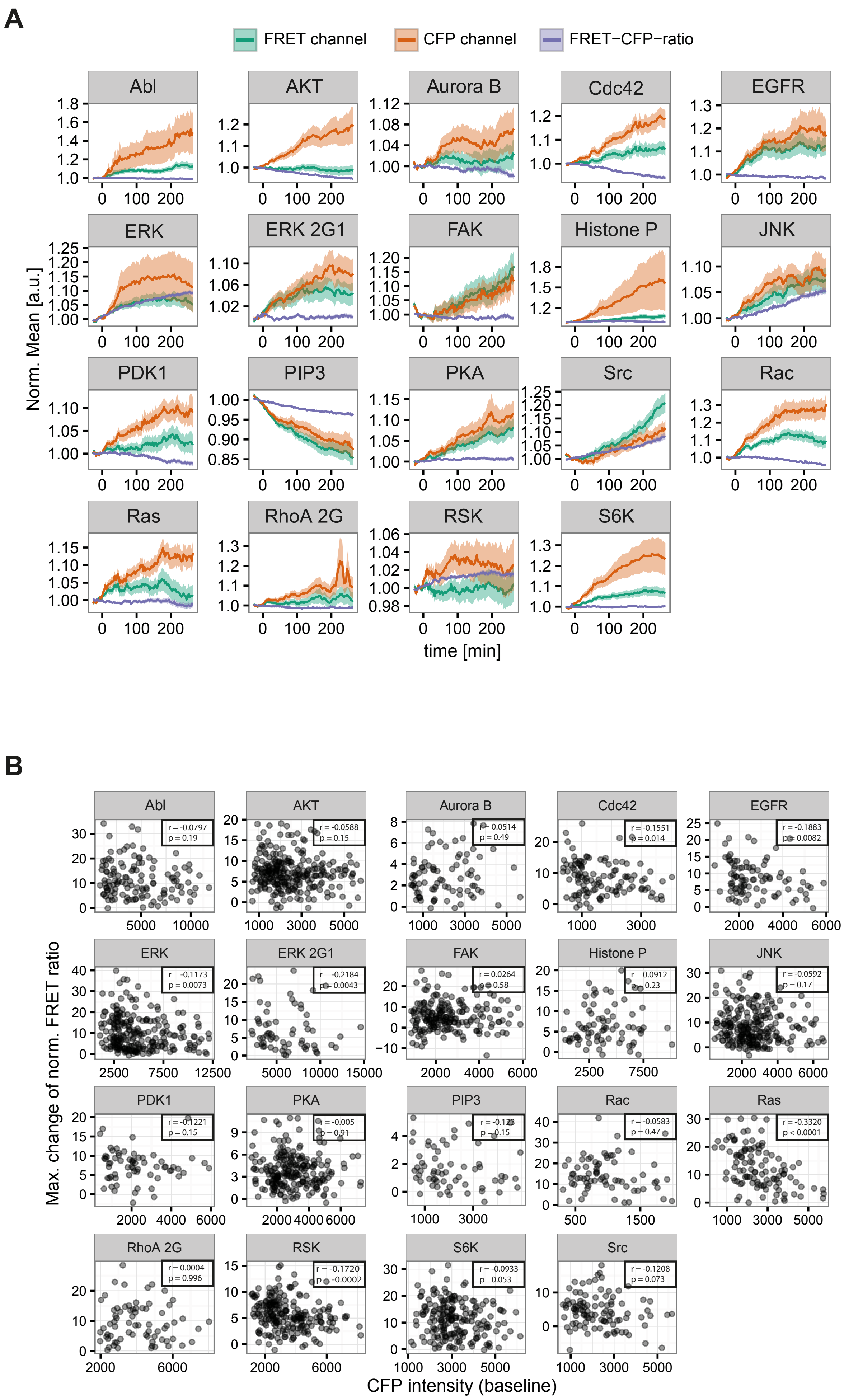

Supplementary Figure 7

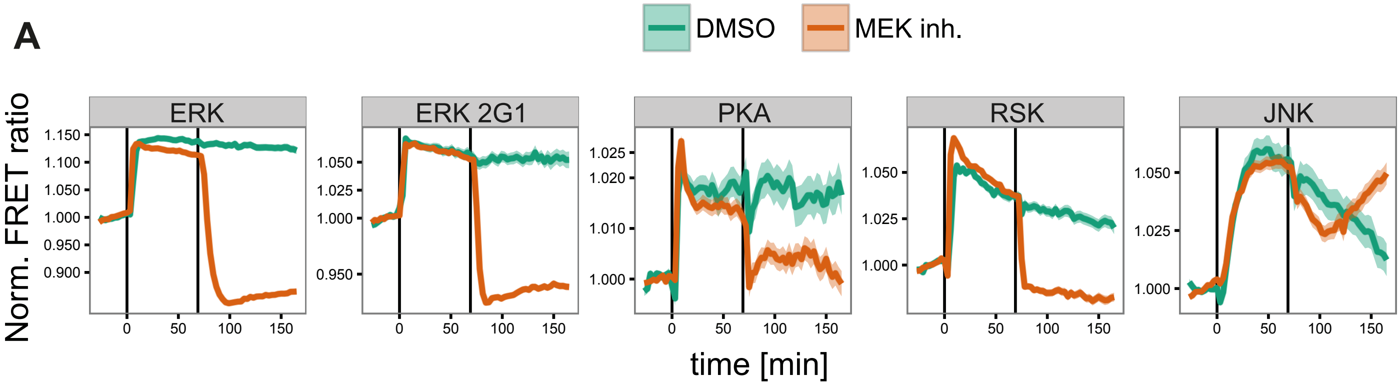

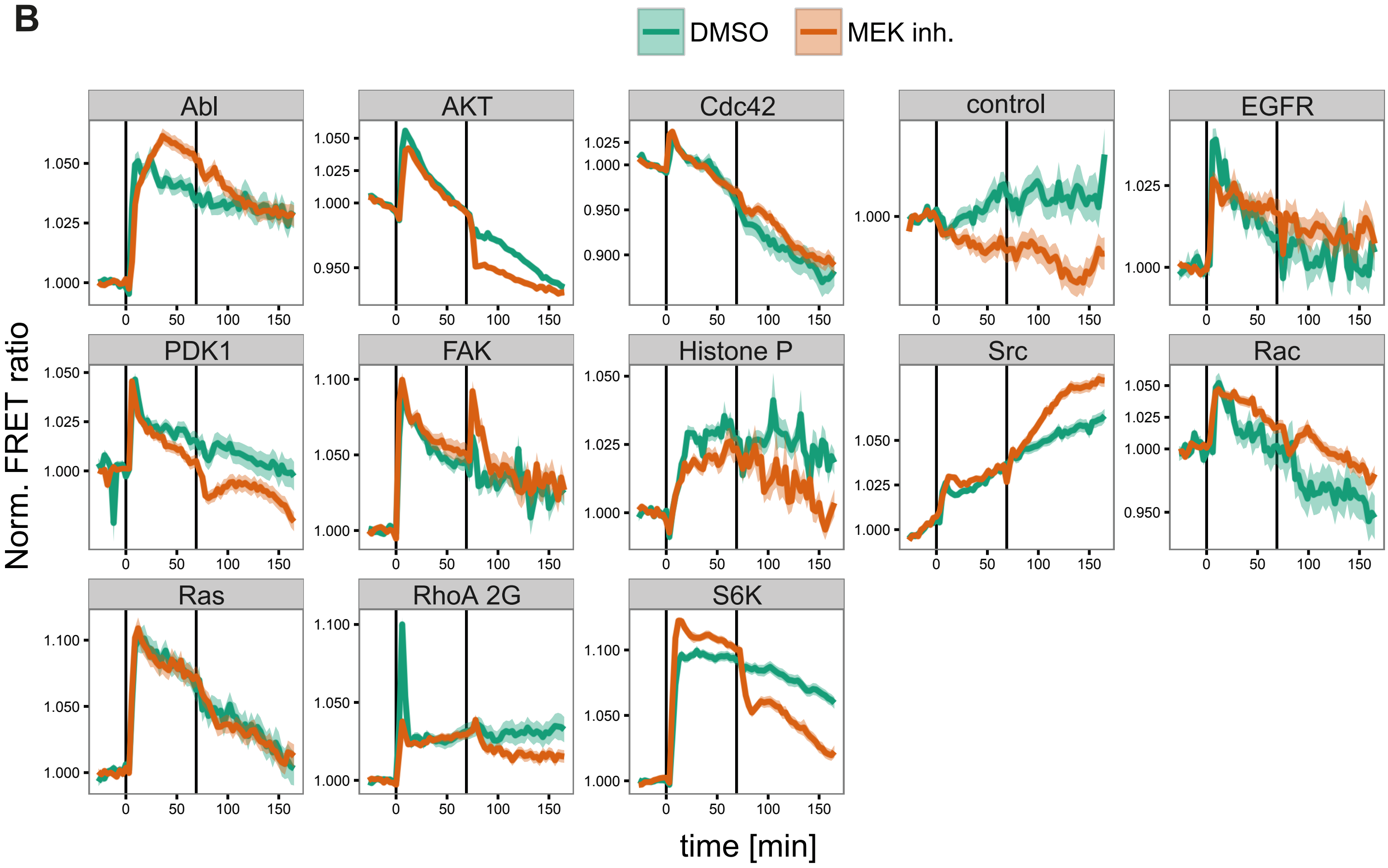

Supplementary Figure 8

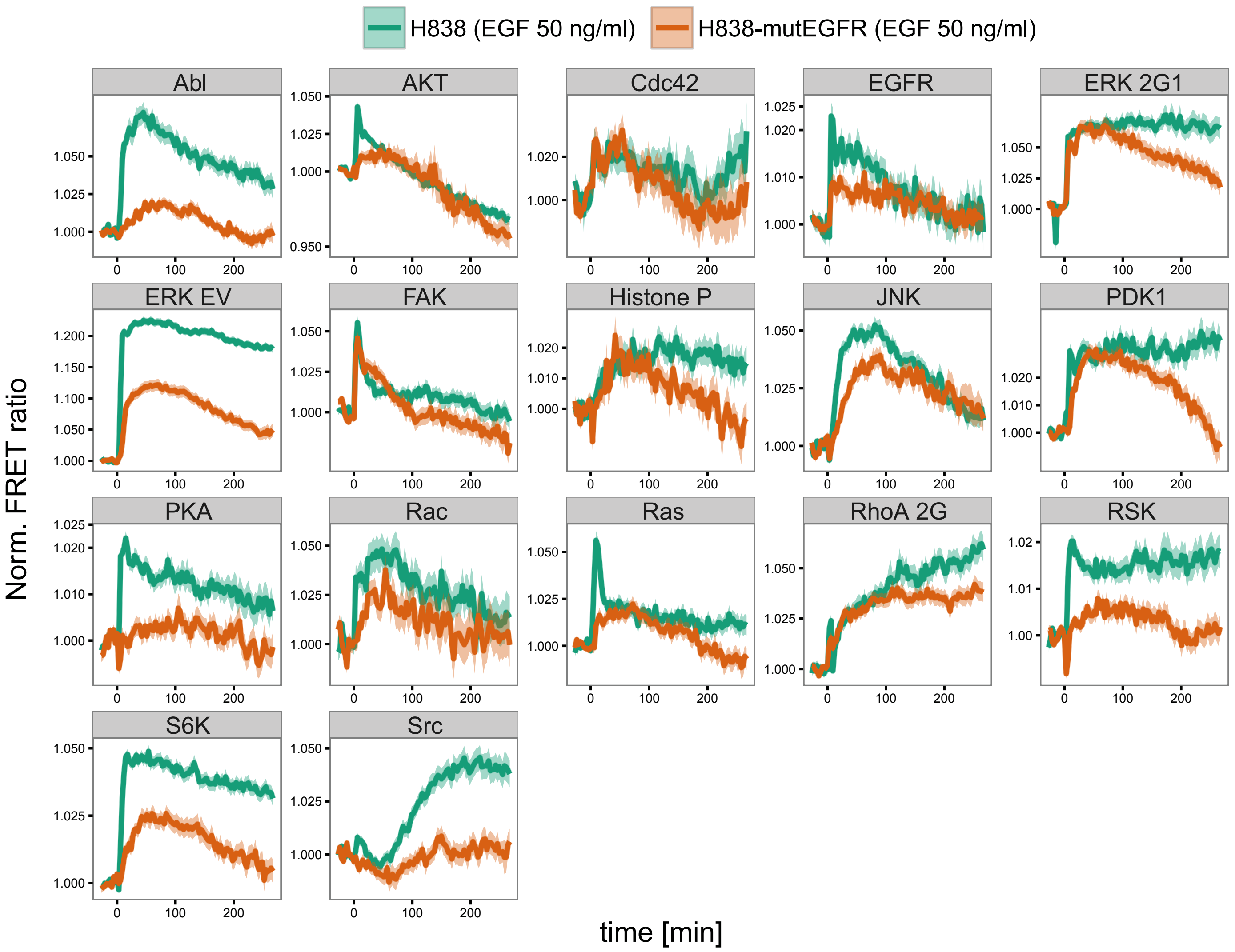

Supplementary Figure 9

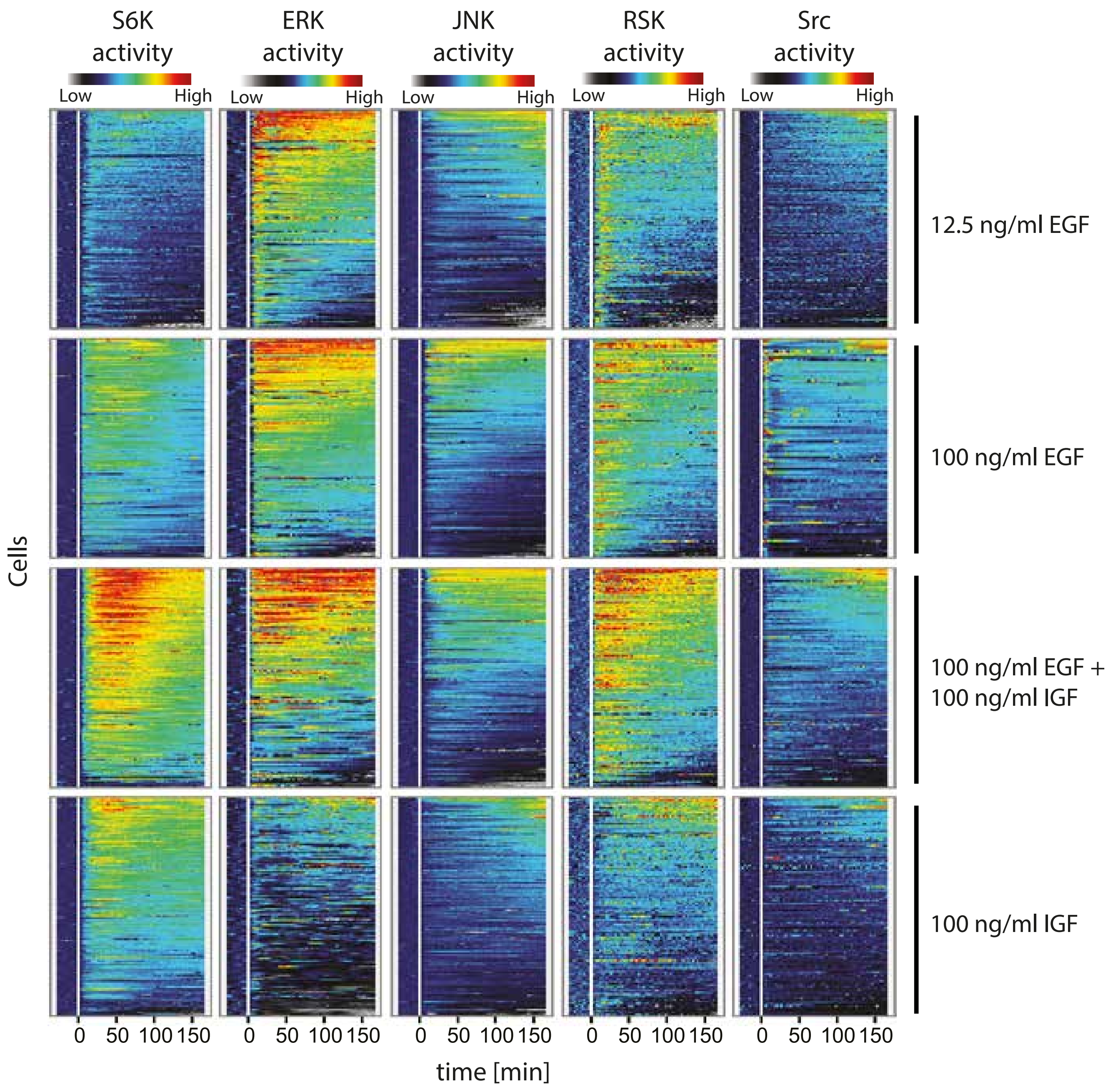

Supplementary Figure 10

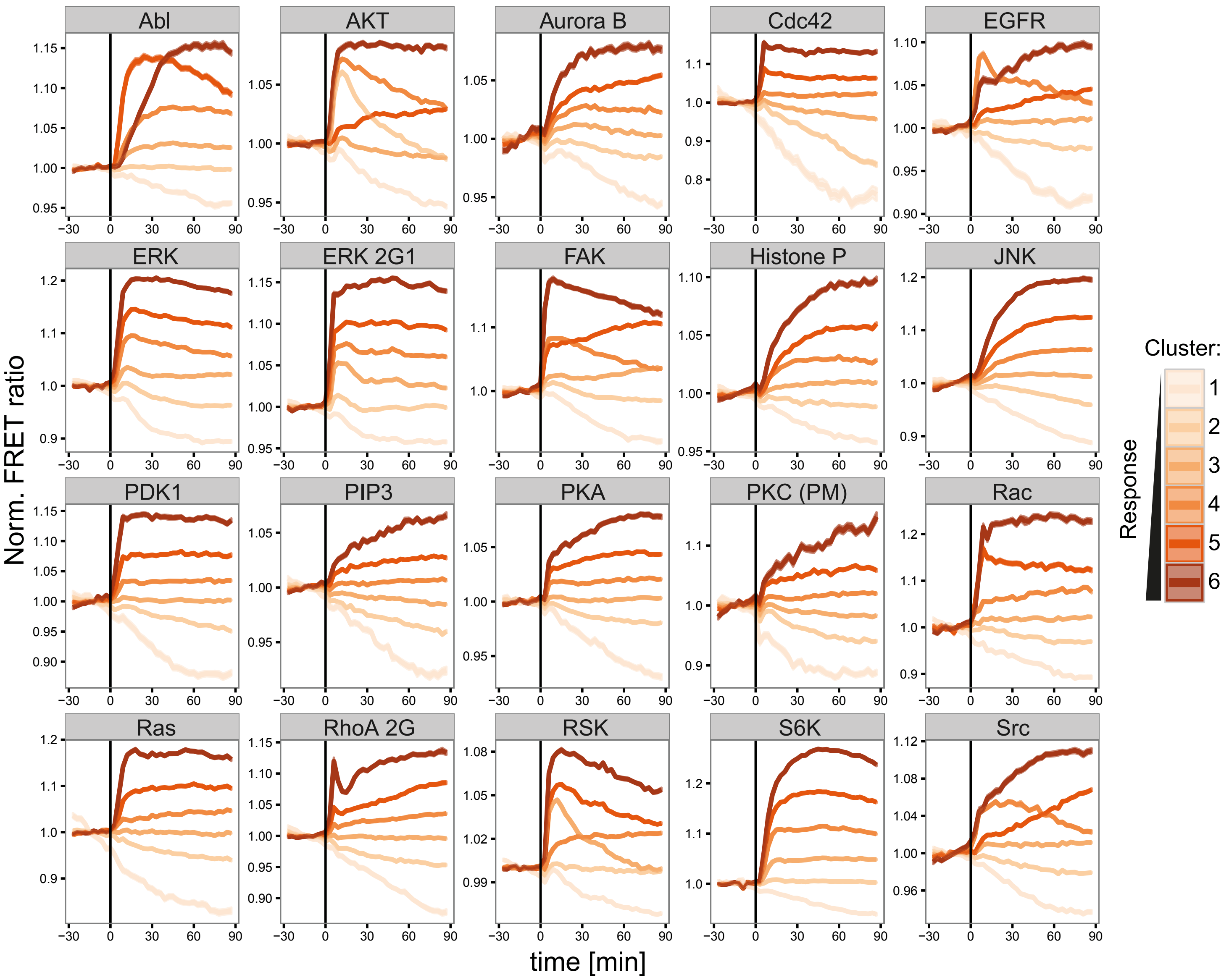

Supplementary Figure 11

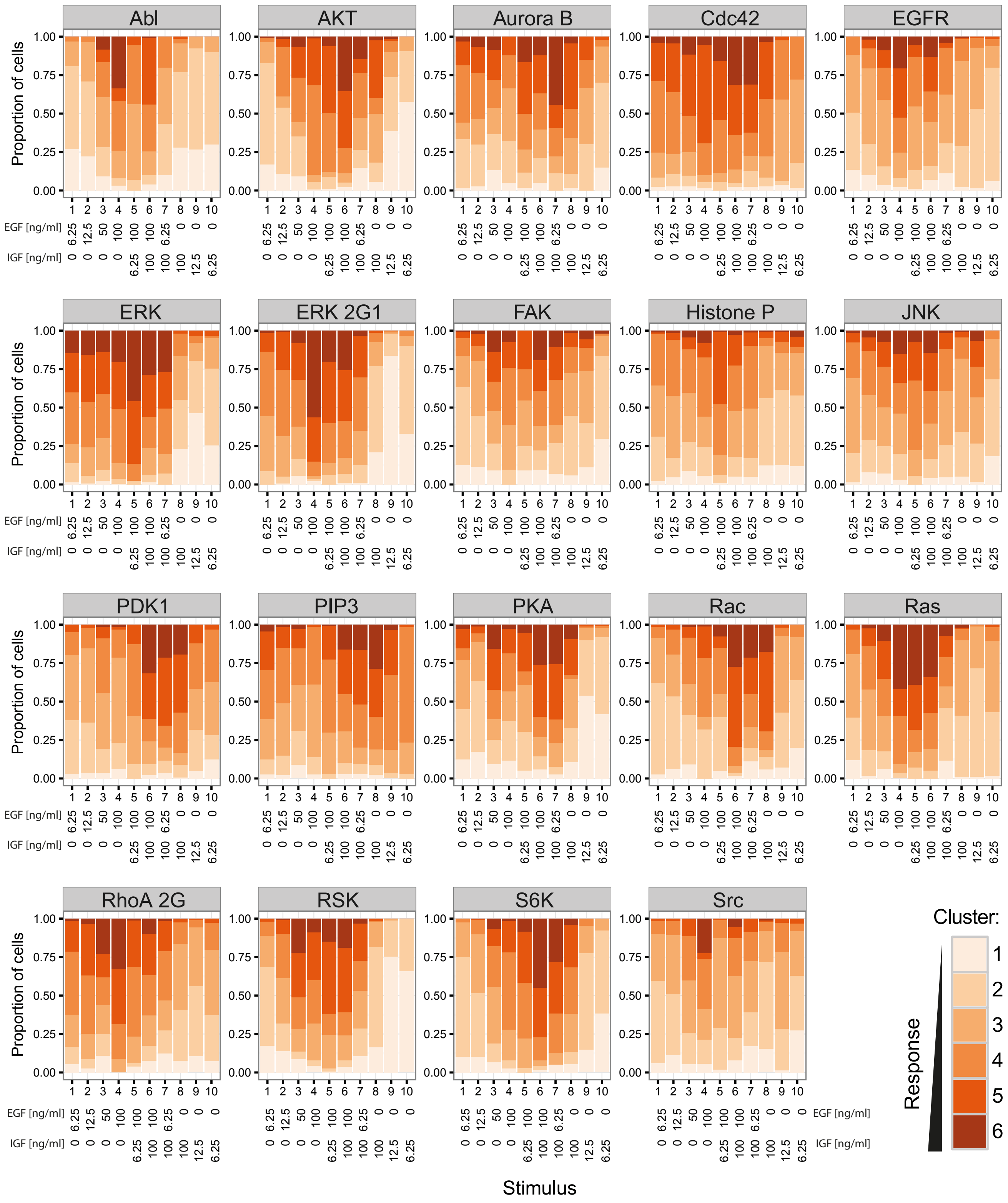

Supplementary Figure 12

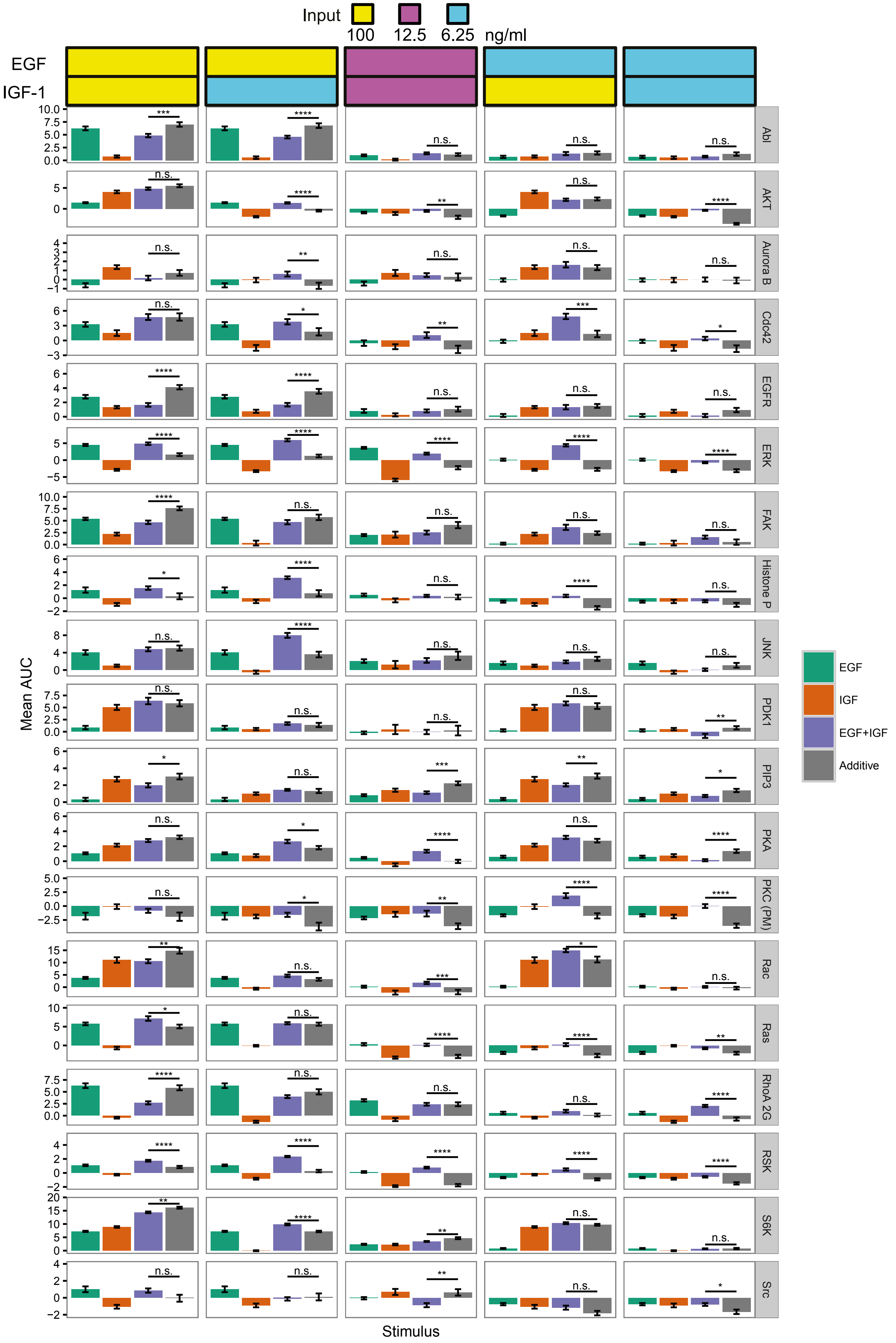

Supplementary Figure 13

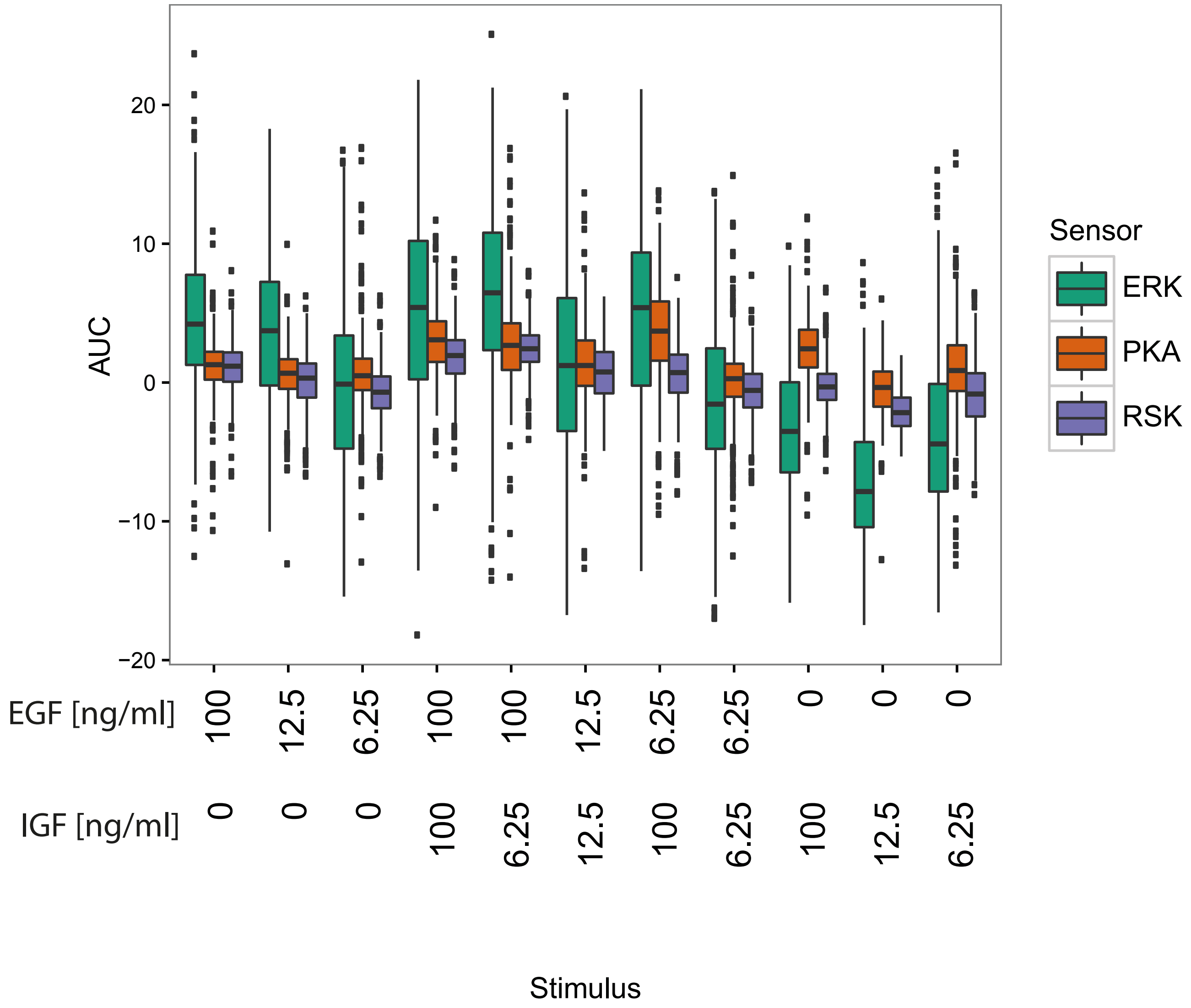

Supplementary Figure 14

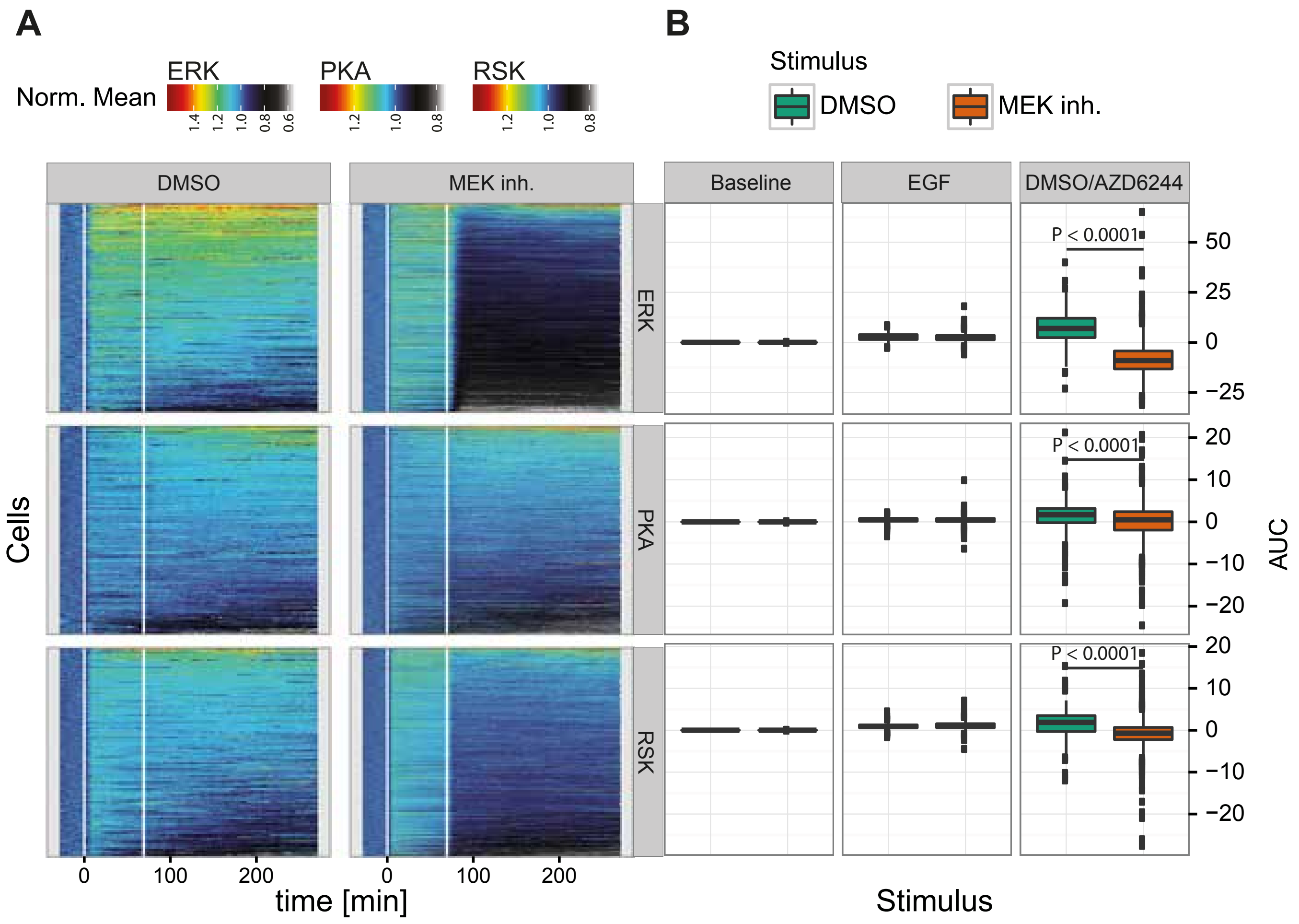

Supplemental Figures and Tables

Figure S1, related to Figure 1

Image analysis pipeline of an in-house developed macro.

Figure S2, related to Figure 1

Compatibility with variety of adherent cells and cross-contamination analysis.

(**A**) Images of cells expressing EKAREV (ERK) FRET biosensor. Scale bar, 200 µm. (**B**) Microarray of HeLa cells printed with plasmids expressing either CFP (blue) or GFP (yellow) in an alternating pattern. Bottom, higher magnification image of four spots from the array. (**C**) Quantification of cells expressing CFP and/or YFP on a spot containing CFP plasmid from figure S2B. Data represent mean of all spots ± SD. (180 spots, three independent experiments).

Figure S3, related to Figure 1

Activity of EGF signaling network in HeLa and H838 cells.

(**A**) FRET biosensors responded to EGF stimulation. HeLa cells were stimulated with 6.25 ng/mL, 12.5 ng/mL, 25 ng/mL or 100 ng/ml EGF at time 0. Data represent mean ± SEM ($n \geq 2$). (**B**) H838 cells were stimulated with 50 ng/mL of EGF at time 0. Data represent mean ± S.E. (n = 4). (**C**) FRET response of H838 cells expressing FRET biosensors after application of EGF (50ng/mL). Ras/ERK/RSK (left) and PDK1/Akt/S6K (right) pathways. Cells were stimulated at time 0. Data represent mean ± SEM (n = 4).

Figure S5, related to Figure 1

Heterogeneity in the response of HeLa cells expressing FRET biosensors upon EGF treatment.

(**A**) Coefficient of variation for each responding FRET biosensors from a single experiment was plotted against time. HeLa cells were stimulated with 100 ng/ml EGF at time 0. (**B**) Randomly selected single cell trajectories from the experiment depicted in Figure S5A.

Figure S6, related to Figure 1

The impact of the FRET biosensor expression level on cellular signaling.

(**A**) Monitoring FRET biosensor expression level and FRET ratio in untreated cells. HeLa cells were stimulated with imaging medium at time 0 to mimic adding of growth factor. FRET biosensor expression levels were approximated to the intensity of donor channel (CFP) before stimulation. Data represent mean ± *SEM* ($n \geq 3$) (**B**) Correlation between the maximum of normalized response and FRET biosensor expression levels. HeLa cells were stimulated with 100 ng/ml EGF ($n > 65$, depending on the FRET biosensor). FRET biosensor expression levels were approximated by averaging 10 frames of donor channel (CFP) before stimulation. r: Kendall's correlation coefficient. p: p-value.

Figure S7, related to Figure 2

Pharmacological perturbation of EGF signaling network activity by a MEK inhibitor (AZD6244).

(**A**) Strongly effected signaling molecules by AZD6244 and (**B**) signaling molecules showing moderate or no effect upon adding AZD6244. HeLa cells were stimulated with EGF (100 ng/mL) at time 0 and treated with DMSO or AZD6244 (5 µM) after 69 min. Data represent mean ± SEM (n = 2, DMSO, and n=3, AZD6244).

Figure S8 , related to Figure 2

Perturbation of EGF signaling by expression of constitutively active EGFR.

H838wt or H838 cells expressing EGFR with both activating L858R and resistant T790M mutations were stimulated with 50 ng/mL of EGF at time 0. Data represent mean ± *SEM* (n = 3).

Figure S9, related to Figure 3

Example of heterogeneous dynamics of protein activity under various conditions in HeLa cells.

Each line of the heatmaps represents a single cell. HeLa cells were treated with 12.5 ng/ml EGF, 100 ng/ml EGF, 100 ng/ml EGF + 100 ng/ml IGF-1, 100 ng/ml IGF-1 at time 0 (vertical white line) (100< n <450).

Figure S10, related to Figure 3

Cluster representative time series.

For each biosensor, normalized to untreated cells time series from all experiments were subjected to K-means clustering with squared euclidian distance. The representative cluster trajectories are color coded. Data represent mean ± *SEM.*

Figure S11, related to Figure 3

Distribution of representative FRET biosensors time series in response to various stimuli.

HeLa cells were treated with 1 – 6.25 ng/ml EGF, 2 – 12.5 ng/ml EGF, 3 – 50 ng/ml EGF, 4 – 100 ng/ml EGF, 5 – 100 ng/ml EGF + 6.25 ng/ml IGF-1, 6 – 100 ng/ml EGF + 100 ng/ml IGF-1, 7 – 6.25 ng/ml EGF + 100 ng/ml IGF-1,8 – 100 ng/ml IGF-1, 9 – 12.5 ng/ml IGF-1,10 – 6.25 ng/ml IGF-1. The clusters are color coded. n > 1296 cells for each biosensor.

Figure S12, related to Figure 4

Difference between calculated (or expected) and experimental additivity.

Area under the curve after stimulation is calculated for each single cell and depicted as mean ± *SEM*. We calculated the expected response (grey) by simple addition of the mean of the area under the curve of “EGF only” and “IGF-1 only” treatments. ****p < 0.0001, ***p < 0.001, **p < 0.01 and *p < 0.05, n.s. ≥ 0.05.

Figure S13, related to Figure 4

PKA activity is correlated with ERK and RSK activities in the presence of EGF.

Area under the curve of single cells plotted against various growth factor treatments. HeLa cells were treated with 6.25 ng/ml EGF, 12.5 ng/ml EGF, 50 ng/ml EGF, 100 ng/ml EGF, 100 ng/ml EGF + 6.25 ng/ml IGF-1, 100 ng/ml EGF + 100 ng/ml IGF-1, 6.25 ng/ml EGF + 100 ng/ml IGF-1, 100 ng/ml IGF-1, 12.5 ng/ml IGF-1, 6.25 ng/ml IGF-1. 100 < n < 542 cells for each biosensor. Horizontal lines indicate the median, boxes the 25th–75th percentile, vertical lines the total range, and (▪) the outliers for each population

Figure S14, related to Figure 2

Single cell analysis of the MEK inhibitor induced decrease in PKA, ERK and RSK activity.

(**A**) Single cell dynamics of PKA, RSK and ERK activity. Data come from figure S7. Each line of the heatmaps represents a single cell. 347 < n < 1000 cells for each biosensor. (**B**) Distribution of ERK, PKA and RSK activity in cells. Area under the curve was calculated for each single cell: before stimulation (baseline), between EGF and DMSO/AZD6244 stimulation and after DMSO/AZD6244. HeLa cells were stimulated with EGF (100 ng/mL) at time 0 and treated with DMSO or AZD6244 (5 μM) after 69 min. Horizontal lines indicate the median, boxes the 25th–75th percentile, vertical lines the total range, and (▪) the outliers for each population

Table S1, related to Figure 1
FRET biosensors used in this study

Target	Sensor Name	Domains	Named In this work	Ref
ERK	EKAREV	WW (phosphopeptide binding domain)-Substrate domain (PDVPRTPVDKAKLSFQFP)	ERK EV	(Komatsu et al., 2011)
ERK	EKAR2G1	WW (phosphopeptide binding domain) - Substrate domain (PDVPRTPVDKAKLSFQFP)	ERK2G1	(Fritz et al., 2013)
RhoA	RhoA2G	RhoA-binding domain (RBD) of rhotekin– RhoA	RhoA	(Fritz et al., 2013)
Ras	RaichuEV-Ras (PM)	H-Ras domain Raf RBD – Ras-binding domain of Raf 1	Ras	(Komatsu et al., 2011)
Rac1	RaichuEV-Rac1 (PM)	PAK CRIB domain - Cdc42/Rac-interactive binding Rac1<br>K-Ras CT domain – targeting to plasma membrane	Rac	(Komatsu et al., 2011)
RSK	Eevee-RSK	FHA1 domain (phosphopeptide binding Ser-1798 of TSC2) - Substrate domain (GQRKRLITSVDDFTE)<br>NES	RSK	(Komatsu et al., 2011)
S6K	Eevee-S6K	FHA1 domain (phosphopeptide binding<br>Thr-1135 of Rictor) - Substrate domain (NRRIRTLTEPDVDFN)<br>NES	S6K	(Komatsu et al., 2011)
Akt	Eevee-Akt	Akt PH domain - FHA1 domain (phosphopeptide binding) - Substrate domain (RKRDRLGTLGD)<br>Akt substrate<br>NES	Akt	(Komatsu et al., 2011)
PKC	Eevee-PKC	PKCβ C1 domain - FHA1 domain (phosphopeptide binding) - Substrate domain (KKKKKRFTFKDSFKL)<br>NES	PKC EV	(Komatsu et al., 2011)
JNK	JNKAR1EV	FHA1-EV- Substrate domain (DSVKTPEDEGNPLLEQLEKK)	JNK	(Komatsu et al., 2011)
EGFR/abl	PicchuEV	CrkII-SH2-SH3 domain-<br>217–225 residues of CrkII	EGFR	(Komatsu et al., 2011)

		(EPGPYAQPS)		
PIP3/PI(3,4)P2	InPAkt pmInPAKt (PM)	PH AKT -pseudoligand	PIP3	(Ananthanarayanan et al., 2005)
Src	Src biosensor	CFP-SH2 (Src)- Substrate (synthetic)	Src	(Ouyang et al., 2008)
Cdc42	Raichu-Cdc42 (PM)	PAK1-Cdc42 (EV linker was cloned in between PAK1 and Cdc42)	Cdc42	(Itoh et al., 2002)
PAK1	Pakabix (PM) Pakabi	human PAK1, Pakabix carries the C-terminal region of Ki-Ras4B	PAK (PM) PAK	(Parrini et al., 2009)
Calcineurin	CaNARI	domain of NFAT1	Calcineurin	(Newman and Zhang, 2008)
FAK	FAK biosensor	SH2(c-Srk) -substrate	FAK	(Seong et al., 2011)
cRaf	Prin-cRaf	cRaf	cRaf	(Terai and Matsuda, 2005)
bRaf	Prin-bRaf	bRaf	bRaf	(Terai and Matsuda, 2006)
Histone H3-K9 methylation	H3 K9 reporter	HP1 Chromodomain-histone H3	Histone K9 Me	(Lin et al., 2004)
Histone H3-K27 methylation	H3 K27 reporter	Polycomb (Pc) Chromodomain-histone H3	Histone K27 Me	(Lin et al., 2004)
Histone H3-S28 phosphorylation	H3 S28 reporter	14-3-3t – 615-644 residues of Cbl	Histone P	(Lin and Ting, 2004)
Cdk1	Cdk1 Sensor	Polo-Box Domain of Plk1-(GGT)5 linker-Cyclin B1 containing the Ser126 autophosphorylation site	Cdk1	(Gavet and Pines, 2010)
PDK1	PARE (PM)	PDK1	PDK1	(Gao et al., 2011)
Caspase-3	Caspase-3 reporter	DEVDR	Caspase-3	(Albeck et al., 2008)
Caspase-8/10	Caspase-8/10 reporter	IETD2x	Caspase-8/10	(Albeck et al., 2008)
PKA	AKAR3EV	FHA1 domain - PKA substrate (LRRATLVD) - NES	PKA	(Komatsu et al., 2011)
PKC	erCKAR (ER) pmCKAR (PM)	FHA2 (Rad53P)-Substrate(synthetic)	PKC (ER)	(Violin et al., 2003)
Rap1	Raichu-Rap1 (PM)	Rap1A-Raf	Rap1	(Mochizuki et al., 2001)
RalA	Raichu-RalA (PM)	RalA-RalBP1	RalA	(Takaya et al., 2004)
RhoA	RhoA	PKN -RhoA	RhoA	(Yoshizaki et al., 2003)
Ca2+	TN-XL	chicken skeletal muscle TnC	Ca2+ (TNXL)	(Mank et al., 2006)

CaMKII	CaMK2α	CaMK2α	Ca2+	(Piljic et al., 2011)
Ca2+	Twitch	TnC domain	Ca2+ (Twitch)	(Thestrup et al., 2014)
Pickles	Abl	CrkL	Abl	(Mizutani et al., 2010)
Aurora B	Aurora B (chromatin)<br>Aurora B (cytosol)<br>Aurora B (centromer)	FHA2-substrate(Kif2 57-70)	Aurora B H2B<br><br>Aurora B<br><br>Aurora B centro	(Fuller et al., 2008)
Control	C32V		control	(Koushik et al., 2006)
Control 2	DEVG	DEVDR mutated to DEVG	DEVG	(Albeck et al., 2008)

Albeck, J.G., Burke, J.M., Spencer, S.L., Lauffenburger, D.A., and Sorger, P.K. (2008). Modeling a snap-action, variable-delay switch controlling extrinsic cell death. PLoS biology *6*, 2831-2852.

Ananthanarayanan, B., Ni, Q., and Zhang, J. (2005). Signal propagation from membrane messengers to nuclear effectors revealed by reporters of phosphoinositide dynamics and Akt activity. Proceedings of the National Academy of Sciences of the United States of America *102*, 15081-15086.

Fritz, R.D., Letzelter, M., Reimann, A., Martin, K., Fusco, L., Ritsma, L., Ponsioen, B., Fluri, E., Schulte-Merker, S., van Rheenen, J., et al. (2013). A versatile toolkit to produce sensitive FRET biosensors to visualize signaling in time and space. Science signaling *6*, rs12.

Fuller, B.G., Lampson, M.A., Foley, E.A., Rosasco-Nitcher, S., Le, K.V., Tobelmann, P., Brautigan, D.L., Stukenberg, P.T., and Kapoor, T.M. (2008). Midzone activation of aurora B in anaphase produces an intracellular phosphorylation gradient. Nature *453*, 1132-1136.

Gao, X., Lowry, P.R., Zhou, X., Depry, C., Wei, Z., Wong, G.W., and Zhang, J. (2011). PI3K/Akt signaling requires spatial compartmentalization in plasma membrane microdomains. Proceedings of the National Academy of Sciences of the United States of America *108*, 14509-14514.

Gavet, O., and Pines, J. (2010). Progressive activation of CyclinB1-Cdk1 coordinates entry to mitosis. Developmental cell *18*, 533-543.

Itoh, R.E., Kurokawa, K., Ohba, Y., Yoshizaki, H., Mochizuki, N., and Matsuda, M. (2002). Activation of rac and cdc42 video imaged by fluorescent resonance energy transfer-based single-molecule probes in the membrane of living cells. Molecular and cellular biology *22*, 6582-6591.

Komatsu, N., Aoki, K., Yamada, M., Yukinaga, H., Fujita, Y., Kamioka, Y., and Matsuda, M. (2011). Development of an optimized backbone of FRET biosensors for kinases and GTPases. Molecular biology of the cell *22*, 4647-4656.

Koushik, S.V., Chen, H., Thaler, C., Puhl, H.L., 3rd, and Vogel, S.S. (2006). Cerulean, Venus, and VenusY67C FRET reference standards. Biophysical journal *91*, L99-L101.

Lin, C.W., Jao, C.Y., and Ting, A.Y. (2004). Genetically encoded fluorescent reporters of histone methylation in living cells. Journal of the American Chemical Society *126*, 5982-5983.

Lin, C.W., and Ting, A.Y. (2004). A genetically encoded fluorescent reporter of histone phosphorylation in living cells. Angewandte Chemie *43*, 2940-2943.

Mank, M., Reiff, D.F., Heim, N., Friedrich, M.W., Borst, A., and Griesbeck, O. (2006). A FRET-based calcium biosensor with fast signal kinetics and high fluorescence change. Biophysical journal *90*, 1790-1796.
Mizutani, T., Kondo, T., Darmanin, S., Tsuda, M., Tanaka, S., Tobiume, M., Asaka, M., and Ohba, Y. (2010). A novel FRET-based biosensor for the measurement of BCR-ABL activity and its response to drugs in living cells. Clinical cancer research : an official journal of the American Association for Cancer Research *16*, 3964-3975.
Mochizuki, N., Yamashita, S., Kurokawa, K., Ohba, Y., Nagai, T., Miyawaki, A., and Matsuda, M. (2001). Spatio-temporal images of growth-factor-induced activation of Ras and Rap1. Nature *411*, 1065-1068.
Newman, R.H., and Zhang, J. (2008). Visualization of phosphatase activity in living cells with a FRET-based calcineurin activity sensor. Molecular bioSystems *4*, 496-501.
Ouyang, M., Sun, J., Chien, S., and Wang, Y. (2008). Determination of hierarchical relationship of Src and Rac at subcellular locations with FRET biosensors. Proceedings of the National Academy of Sciences of the United States of America *105*, 14353-14358.
Parrini, M.C., Camonis, J., Matsuda, M., and de Gunzburg, J. (2009). Dissecting activation of the PAK1 kinase at protrusions in living cells. The Journal of biological chemistry *284*, 24133-24143.
Piljic, A., de Diego, I., Wilmanns, M., and Schultz, C. (2011). Rapid development of genetically encoded FRET reporters. ACS chemical biology *6*, 685-691.
Seong, J., Ouyang, M., Kim, T., Sun, J., Wen, P.C., Lu, S., Zhuo, Y., Llewellyn, N.M., Schlaepfer, D.D., Guan, J.L., et al. (2011). Detection of focal adhesion kinase activation at membrane microdomains by fluorescence resonance energy transfer. Nature communications *2*, 406.
Takaya, A., Ohba, Y., Kurokawa, K., and Matsuda, M. (2004). RalA activation at nascent lamellipodia of epidermal growth factor-stimulated Cos7 cells and migrating Madin-Darby canine kidney cells. Molecular biology of the cell *15*, 2549-2557.
Terai, K., and Matsuda, M. (2005). Ras binding opens c-Raf to expose the docking site for mitogen-activated protein kinase kinase. EMBO reports *6*, 251-255.
Terai, K., and Matsuda, M. (2006). The amino-terminal B-Raf-specific region mediates calcium-dependent homo- and hetero-dimerization of Raf. The EMBO journal *25*, 3556-3564.
Thestrup, T., Litzlbauer, J., Bartholomaus, I., Mues, M., Russo, L., Dana, H., Kovalchuk, Y., Liang, Y., Kalamakis, G., Laukat, Y., et al. (2014). Optimized ratiometric calcium sensors for functional in vivo imaging of neurons and T lymphocytes. Nat Methods *11*, 175-182.
Violin, J.D., Zhang, J., Tsien, R.Y., and Newton, A.C. (2003). A genetically encoded fluorescent reporter reveals oscillatory phosphorylation by protein kinase C. The Journal of cell biology *161*, 899-909.
Yoshizaki, H., Ohba, Y., Kurokawa, K., Itoh, R.E., Nakamura, T., Mochizuki, N., Nagashima, K., and Matsuda, M. (2003). Activity of Rho-family GTPases during cell division as visualized with FRET-based probes. The Journal of cell biology *162*, 223-232.
