# Supplementary material for: High-Content Imaging Platform for Profiling Intracellular Signaling Network Activity in Living Cells
Source: Cell Chem Biol. 2016 Dec 22;23(12):1550–9. doi: 10.1016/j.chembiol.2016.11.008 (PMC5193178; doi:10.1016/j.chembiol.2016.11.008)
Supplement: Document S2. Article plus Supplemental Information [file mmc2.pdf]

Resource

Cell Chemical Biology

High-Content Imaging Platform for Profiling Intracellular Signaling Network Activity in Living Cells

Graphical Abstract

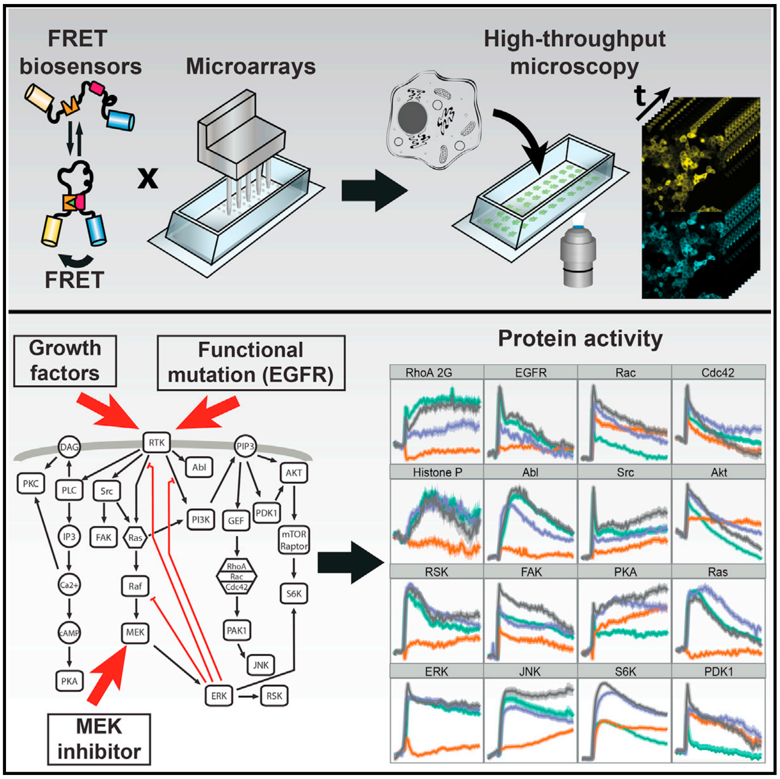

Highlights

- A FRET biosensor-based array platform for monitoring signaling network activity
- The platform enables multi-dimensional data generation in a single experiment
- The effect of perturbing the network by drugs is demonstrated
- Crosstalk of two growth factors produces distinct activity patterns

Authors

Dmitry Kuchenov, Vibor Laketa, Frank Stein, Florian Salopiata, Ursula Klingmüller, Carsten Schultz

Correspondence

schultz@embl.de

In Brief

Kuchenov et al. describe a FRET-based multi-parameter imaging platform (FMIP) that allows simultaneous high-throughput monitoring of multiple signaling pathways. The authors employ FMIP to monitor crosstalk between EGFR and IGF-1R signaling, signaling perturbations caused by EGFR mutations, and the effects of selumetinib on the EGFR network.

Kuchenov et al., 2016, Cell Chemical Biology *23*, 1550–1559
December 22, 2016 © 2016 The Authors. Published by Elsevier Ltd.
http://dx.doi.org/10.1016/j.chembiol.2016.11.008

Please cite this article as: Kuchenov et al., High-Content Imaging Platform for Profiling Intracellular Signaling Network Activity in Living Cells, Cell Chemical Biology (2016), http://dx.doi.org/10.1016/j.chembiol.2016.11.008

CellPress

Cell Chemical Biology
Resource

High-Content Imaging Platform for Profiling Intracellular Signaling Network Activity in Living Cells

Dmitry Kuchenov,[1] Vibor Laketa,[1,5] Frank Stein,[1] Florian Salopiata,[2,3] Ursula Klingmüller,[2,3] and Carsten Schultz[1,3,4,6,*]
[1]Cell Biology and Biophysics Unit, European Molecular Biology Laboratory (EMBL), 69117 Heidelberg, Germany
[2]Division of Systems Biology of Signal Transduction, Systems Biology of Signal Transduction, German Cancer Research Center (DKFZ), 69120 Heidelberg, Germany
[3]Translational Lung Research Center (TLRC), Member of the German Center for Lung Research (DZL), 69120 Heidelberg, Germany
[4]Department of Physiology and Pharmacology, Oregon Health and Science University, Portland, OR 97201, USA
[5]Present address: Department of Infectious Diseases, Virology, University Hospital Heidelberg, 69120 Heidelberg, Germany
[6]Lead Contact
*Correspondence: schultz@embl.de
http://dx.doi.org/10.1016/j.chembiol.2016.11.008

SUMMARY

Essential characteristics of cellular signaling networks include a complex interconnected architecture and temporal dynamics of protein activity. The latter can be monitored by Förster resonance energy transfer (FRET) biosensors at a single-live-cell level with high temporal resolution. However, these experiments are typically limited to the use of a couple of FRET biosensors. Here, we describe a FRET-based multi-parameter imaging platform (FMIP) that allows simultaneous high-throughput monitoring of multiple signaling pathways. We apply FMIP to monitor the crosstalk between epidermal growth factor receptor (EGFR) and insulin-like growth factor-1 receptor signaling, signaling perturbations caused by pathophysiologically relevant EGFR mutations, and the effects of a clinically important MEK inhibitor (selumetinib) on the EGFR network. We expect that in the future the platform will be applied to develop comprehensive models of signaling networks and will help to investigate the mechanism of action as well as side effects of therapeutic treatments.

INTRODUCTION

Intracellular signaling networks are complex machineries that reliably receive and process extracellular information to adjust the physiological state of cells to environmental changes. The signaling networks are formed by the interplay of hundreds of proteins and second messengers (ions, lipids, triphosphates, etc.) featuring an extensively branched and feedback-based architecture (Kholodenko et al., 2010; Natarajan et al., 2006; Pawson, 2004). Recently, it was established that signaling networks have a remarkable ability to encode the identity and quantity of a given stimulus by temporal patterns and/or dynamics of individual signaling components within the network architecture (Kholodenko et al., 2010; Kubota et al., 2012; Purvis and Lahav, 2013; Toettcher et al., 2013). These signaling network characteristics result in precise control over cellular responses and the ability to adapt to various perturbations. The prediction of the latter is of great interest for basic and translational research. A better understanding of signaling network operation will allow manipulating key parameters with higher precision and therefore facilitate the development of new pharmacological strategies. To understand how cellular signaling networks integrate information from the extracellular environment, how they evoke specific cellular responses and, most importantly, how normal signaling is rewired under the course of a disease, a method is needed that allows comprehensive system-level analysis of multiple signaling pathway dynamics under identical conditions. Such a toolbox should be able to detect second messengers as well as key protein activities of the network with high temporal resolution, and also clarify the interplay between signaling events and phenotypic changes.

Current high-throughput approaches to measure activation of a signaling network at a single-cell level include flow and mass cytometry, microfluidics, single-cell western blot, and fluorescence lifetime imaging microscopy on cell arrays (CA-FLIM) (Bendall et al., 2012; Cheong et al., 2009; Giesen et al., 2014; Grecco et al., 2010; Hughes et al., 2014). Although these single-cell methods provide advantages such as multiplexed quantification, they all suffer from several drawbacks. Most of the methods are based on endpoint measurement of post-translational modifications (PTMs) resulting in limited information on dynamic changes. The second challenge is their dependence on highly specific antibodies because cross-reactivity induces misleading signals in complex biological backgrounds, for which it is difficult to correct (Stadler et al., 2013). The third challenge is to employ a single phosphorylation state or another PTM as an approximation for protein activity, which is not necessarily the case (de la Cruz-Herrera et al., 2015; Liu et al., 2014). Finally, flow cytometry only works with a suspension of cells. Placing inherently adherent cells in a non-natural, suspension environment likely alters the activity of a signaling network and makes it difficult to relate signaling events to a phenotypic difference.

In this study, we describe a Förster resonance energy transfer (FRET)-based multi-parameter imaging platform (FMIP) that allows monitoring of the activity of multiple signaling pathways in

This is an open access article under the CC BY license (http://creativecommons.org/licenses/by/4.0/).

adherent single live cells with high temporal resolution. The FMIP exploits publicly available FRET biosensors, uses conventional microscopes and, by taking advantage of cell microarrays (Ziauddin and Sabatini, 2001), is able to image up to 384 FRET sensors in a single experiment. To illustrate the potential of the platform, we used 40 FRET biosensors in a single experiment and we profiled a cancer-relevant signaling network by monitoring: (1) the perturbation of epidermal growth factor receptor (EGFR) signaling caused by the activating mutation L858R and the resistance mutation T790M of EGFR, (2) the effects of an MEK inhibitor on EGFR network activity; and (3) the crosstalk of the EGFR and insulin-like growth factor-1 receptor (IGF-1R) signaling networks.

RESULTS

Design of a FRET-Based Multi-parameter Imaging Platform

FRET biosensors are used to measure the conformational change of proteins reflecting protein-protein interaction, post-translational modification, concentration of second messengers and, most importantly, protein activities, not merely abundance (Newman et al., 2011). As FRET biosensors measure protein activity at the single-live-cell level in real-time, they offer information on signaling network dynamics. Importantly, FRET biosensors can be successfully used in high-throughput experiments (Bakal et al., 2008). Thus, we reasoned that FRET biosensors are an ideal tool for monitoring multiple signaling events simultaneously in a high-throughput manner.

By combining time-resolved single-live-cell imaging with advances in cell microarrays (Piljic et al., 2011; Ziauddin and Sabatini, 2001) and FRET biosensor technologies, we developed an FMIP to analyze the dynamics of various signaling pathways in real-time. The FMIP employs Lab-Tek chambers printed with 384 spots (200 μm diameter) in a grid-like manner. Each spot contains a plasmid encoding one of 40 different FRET biosensors (Table S1), in replicates (Figure 1A). Adherent mammalian cells are seeded on Lab-Tek chambers pre-printed with FRET biosensors and incubated for 48–72 hr. Each individual spot out of 139 contained cells expressing one of the 40 different FRET biosensors. These are imaged every 3 min with an automated microscope (Figure 1A). Subsequently, image segmentation and intensity measurements are carried out automatically by an in-house developed Fiji macro (Figure S1). The method allows time-resolved monitoring of signaling network dynamics at the single-cell level by taking ratiometric images of the 139 spots within 3 min. Less spots would permit faster data acquisition for each spot. Compared with single-FRET sensor imaging in isolated dishes, FMIP facilitates reproducibility since all cells are placed in a single dish under identical conditions. In comparison with multiplexed expression of FRET biosensors, the FMIP is also less affected by perturbations of the introduced FRET biosensors (Miyawaki, 2003) as each transfected cell expresses only one construct.

Using a slightly modified reverse-transfection protocol described previously (Piljic et al., 2011) (Figure 1A), we successfully transfected different cell lines such as HeLa, H838, C2BBe1, and MIN6 cells (Figure S2A), demonstrating that the FMIP method is applicable to various cultured cell lines. The transfection rates were sufficient to monitor an average of 27–150 cells (depending on the cell line and FRET biosensor) in a single experiment enabling single-cell variation analysis.

To assess whether cells within one spot would become contaminated with plasmids from neighboring spots or cross-contaminated due to cell migration, we printed two plasmids encoding enhanced CFP (ECFP) and EGFP in an alternating pattern. The fluorescence images showed no significant ECFP expression on EGFP-transfected spots and, vice versa, demonstrating clear separation of transfected cells to the respective spots (Figures S2B and S2C).

Next, we determined if the FMIP platform could reliably report time-dependent changes in the well-studied epidermal growth factor (EGF) signaling network (Blagoev et al., 2004; Lemmon and Schlessinger, 2010; Wagner et al., 2013). As expected, EGF triggered activation of Ras-ERK and PDK1-Akt-S6K pathways in HeLa cells (Figures 1B, S3A, and S3B) as well as in H838 cells (Figures S4A and S4B) in a concentration-dependent manner. We used the resulting profile of normalized FRET ratio values as a fingerprint of dynamic responses to hierarchically cluster the time courses of protein activity after EGF addition, which induced distinct kinetics of protein activation. The protein activity dynamics clearly segregated into three clusters: strong, middle, and weak (Figure 1C). The low average coefficient of variation (CV) across replicate experiments over time (7.3%) confirmed the very high data reproducibility. However, not unexpected, we observed much higher variability (depending on the FRET biosensor, CV < 30%) between individual cells within the same experiment (Figures S5A and S5B) for a responding FRET sensor. The experiment reproduced a broad range of observations from previously published studies (Fujita et al., 2014; Wagner et al., 2013), demonstrating its ability to examine the dynamics of the signaling network by monitoring activities of 40 signaling events in parallel.

To test whether biosensor overexpression perturbed the signaling network under observation, we evaluated the effect of FRET biosensor expression in response to EGF. We therefore recorded the change of the FRET biosensor expression level in untreated HeLa cells over time (Figure S6). Although in most cases, the intensity of CFP and the FRET channel emission was increased, the FRET ratio was much more stable demonstrating the advantage of using ratiometric FRET biosensors in general as they provide an internal control for the expression level when the ratio of the FRET channel over the CFP channel is used (Thestrup et al., 2014). Moreover, we observed a very weak or no correlation, except for a Ras FRET biosensor ($r = -0.33$, $p < 0.0001$), between the maximal normalized FRET ratio value and the expression level of the FRET biosensors (Figure S6B). Overall, the data suggest that the assay provides a robust and reproducible readout of signaling network activity at the single-cell level.

Monitoring Perturbations of EGFR Signaling Network Activity

Functional mutations in the cellular genome causing aberrant signaling network activity have been implicated in the progression of various diseases including cancer. Therapies that counteract the downstream effects of the mutation may reverse the course of the disease. To evaluate FMIP in such applications,

Please cite this article as: Kuchenov et al., High-Content Imaging Platform for Profiling Intracellular Signaling Network Activity in Living Cells, Cell Chemical Biology (2016), http://dx.doi.org/10.1016/j.chembiol.2016.11.008

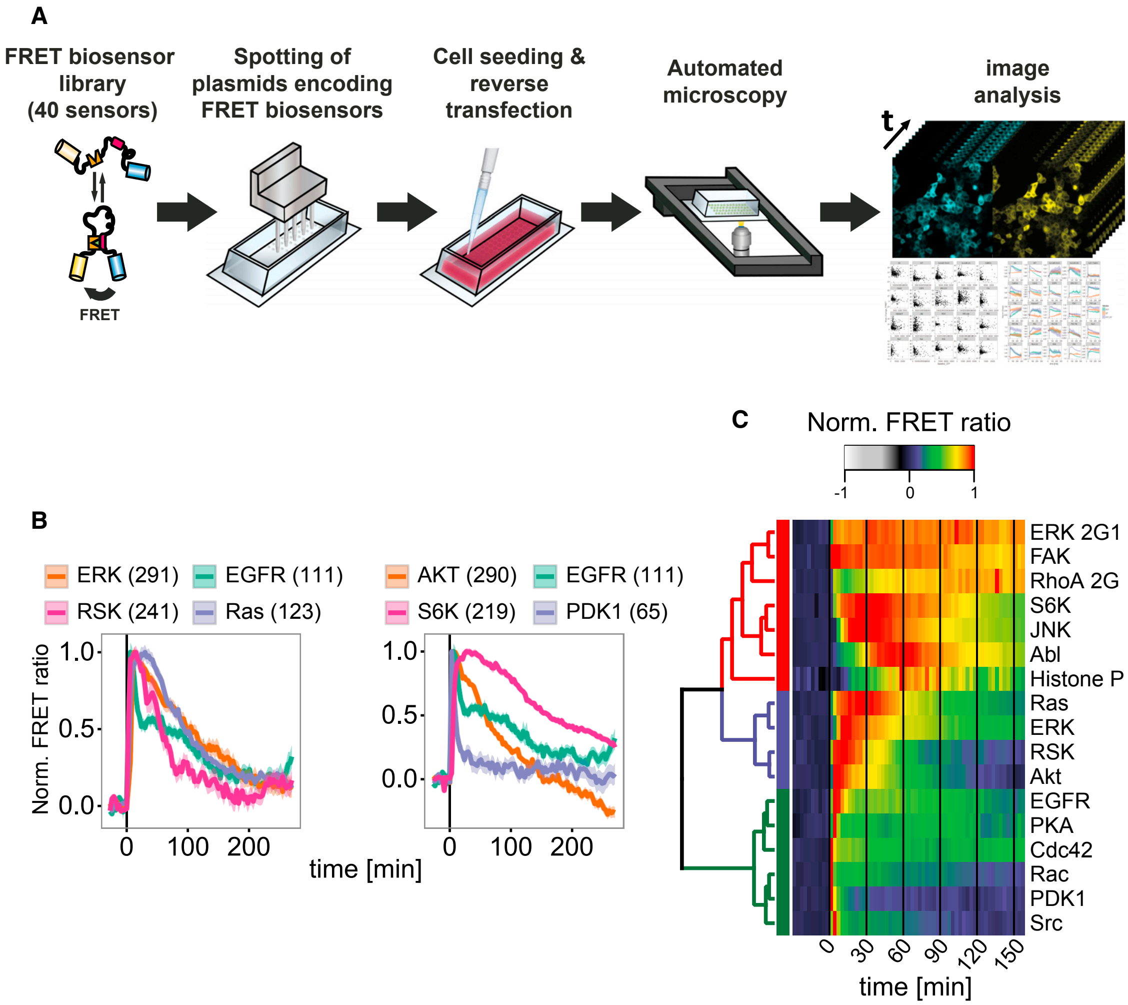

Figure 1. Design and Characterization of the FRET-Based Multi-Parameter Imaging Platform

(A) Method workflow.

(B) Representative FRET ratio traces in HeLa cells expressing sensors for monitoring Ras/ERK/RSK (left) and PDK1/Akt/S6K (right) pathways. Cells were stimulated with EGF (100 ng/mL) at time 0. Data represent mean ± SEM of four independent experiments. The number of cells analyzed is given in parentheses.

(C) Hierarchical clustering of the EGF (100 ng/mL) response amplitude over time. Clusters of responses are color-coded: strong (red), middle (blue), and weak (green). For each FRET biosensor the mean of four independent experiments is represented.

the activity of the EGF signaling network was pharmacologically perturbed in HeLa cells using a clinically relevant MEK inhibitor (selumetinib or AZD6244) (Bekaii-Saab et al., 2011). As predicted, addition of AZD6244 completely abrogated the activity of ERK and RSK (Figures 2A and S7A). In contrast, the activity of Ras, Cdc42, EGFR and FAK was not affected (Figure S7B). Surprisingly, AZD6244 exhibited a strong effect on PKA activity (Figure S7A) and modest effects on Akt, PDK1, S6K, JNK, and Src activities (Figure S7B). The results suggest two scenarios: (1) potential off-target effects of the inhibitor and/or (2) an unpredicted topology of the EGF signaling network. These observations demonstrate that FMIP is a powerful technique that may become highly relevant in the drug discovery process exploring drug targets and off-targets, as well as mechanisms of action across multiple disease settings.

To analyze how pathophysiologically relevant EGFR mutations perturb the signaling network, we profiled EGF signaling activity in lung cancer metastases-derived H838 cells expressing the wild-type EGFR and compared the results to H838 cells harboring the exogenously expressed EGFR that carries the activating mutation L858R and the resistance mutation T790M (H838-EGFRmut). We observed a strong decrease in activity of kinases such as Src, Abl, PKA, and RSK upon stimulation with EGF and modest reduction of Akt, ERK, S6K, PDK1, and JNK activities in H838-EGFRmut cells compared with H838 wild-type cells (Figures 2B and S8). To compare global response patterns between cells expressing EGFRmut and wild-type EGFR, we subjected the obtained EGF signaling data from HeLa (EGFRwt), H838 (EGFRwt), H838 exogenously expressing EGFRmut, and H1975 endogenously expressing EGFRmut cells to principal component analysis (PCA) (Figure 2C). The latter indicates that the global EGF signaling dynamics in H838-EGFRmut and H1975 were clustered together indicating the strong similarity of endogenously and exogenously expressing EGFRmut cells.

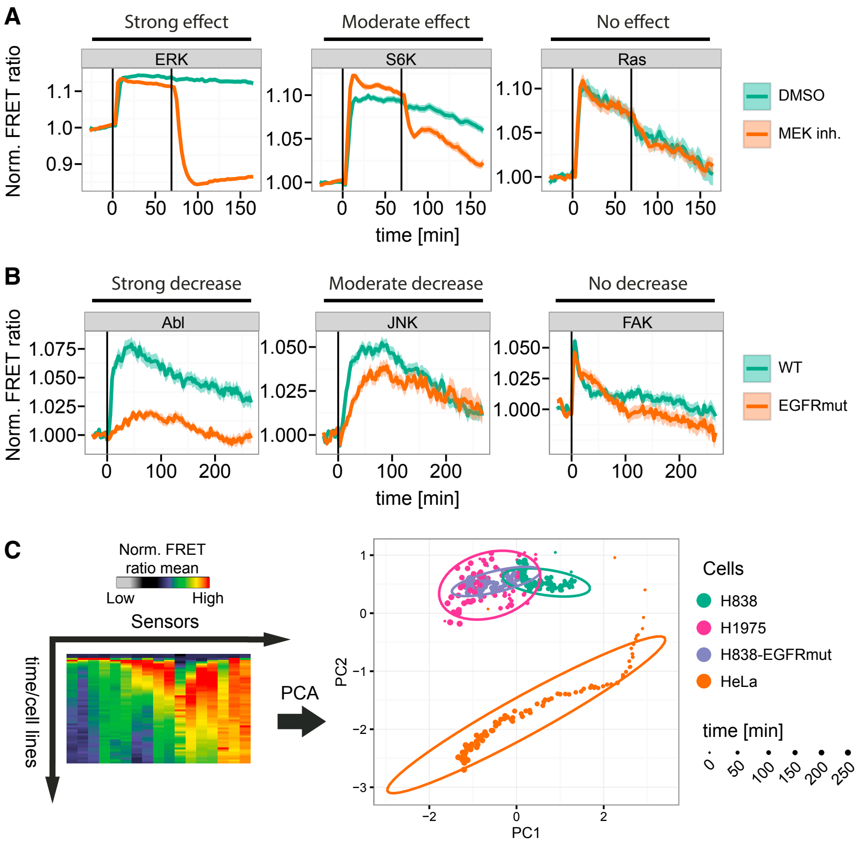

Figure 2. Monitoring Perturbations of the EGF Signaling Network

(A) Pharmacological perturbation of EGF signaling network activity by the MEK inhibitor AZD6244. HeLa cells were stimulated with EGF (100 ng/mL) at time 0 and treated with DMSO or MEK inhibitor (5 μM) after 69 min. Data represent mean ± SEM of two (DMSO) or three (MEK inh.) independent experiments. Representative cases are shown.

(B) Perturbation of EGF signaling by expression of constitutively active EGFR. H838wt or H838-EGFRmut cells exogenously expressing the EGFR that carries activating L858R and resistant T790M mutations were stimulated with 50 ng/mL of EGF at time 0. Representative cases are shown. Data represent mean ± SEM (n = 3).

(C) Principal component analysis of the average EGF response in different cell lines. Each dot represents a single time point while the ovals indicate 70% of time points of the same treatment. Cells were treated with 50 ng/mL EGF.

Interestingly, we observed strong differences between global responses in HeLa and H838 cells suggesting cell line-dependent differences (Figure 2C). Overall, the FRET biosensors in H838-EGFRmut cells responded to EGF with a much altered dynamic pattern in comparison with wild-type H838, providing insight into the changes of this signaling network in response to disease-causing and network-perturbing mutations. Such analysis may form the basis for the unbiased profiling of the clinically observed mutations for their ability to perturb various signaling networks as well as to quantitatively assess the degree of such perturbation, providing information relevant for disease progression, prognosis, design of therapeutic treatment, and drug discovery.

Monitoring the Crosstalk of the EGFR and IGF-1R Signaling Networks

Despite a detailed characterization of signal transduction induced by a single growth factor, it is not well understood how cells integrate and process information of multiple stimuli (Borisov et al., 2009; Worster et al., 2012), which is a physiologically common scenario. We therefore used FMIP to investigate unique and redundant features of EGFR and IGF-1R signaling networks. By using the FMIP platform, we monitored the signaling network in a time-dependent fashion after stimulation with EGF only, IGF-1 only, or after co-stimulation using varying concentrations of growth factors. At the single-cell level, we found that the FRET biosensor response was highly variable across all conditions (Figure S9). Notably, we identified pulsatile activity of ERK (12.5 ng/mL EGF) and Src (100 ng/mL EGF) in some cells that would have been very difficult to obtain using population or/and endpoint assays (Figure S9).

To study signaling network activity at the single-cell level, we combined all single-cell trajectories for each FRET biosensor from across all treatments, on average 2,000 cells per biosensor, and subjected the data to K-means clustering to estimate six representative dynamic patterns (Figure S10). We used the proportion of cells in these clusters as unique signatures of dynamic activity (Figures 3A–3E and S10). In agreement with previous work (Wagner et al., 2013), we observed stronger activation of the PDK1/Akt/S6K pathway by IGF-1 than by EGF (Figures 3A and 3E). In contrast, EGF is a much stronger activator of the Ras/ERK/RSK and Src/FAK pathways. Notably, co-stimulation of the cells with EGF and IGF-1 showed concentration-dependent features that are characteristic signatures of “EGF only” and “IGF-1 only” treatments (Figures 3A–3E). Importantly, the response of the RhoA 2G biosensor suggested decreased activity of RhoA upon co-stimulation, correlating with the findings obtained in another cell type (Novakofski et al., 2009) (Figure S11). Surprisingly, we also observed decreased response of the PIP_3 and Abl FRET biosensors upon co-treatment with EGF and IGF-1, suggesting crosstalk between EGF and IGF-1 signaling. We further subjected the data to PCA. We found that EGF, IGF-1, and combined treatments were clearly clustered (Figure 3F). The PCA indicated that “IGF-1 only” treatment was most separated from “EGF only” stimulation, whereas combined treatments moved the cluster in between “EGF only” and “IGF-1 only.” Strikingly, depending on the ratio of the growth factors the cluster is shifted toward the direction of the growth factor with the higher concentration (Figure 3F). However, the shift in the PCA space is not proportional to the ratio of the growth factors suggesting interaction (synergy and/or antagonism) between EGF and IGF-1. Overall these observations indicate strong crosstalk and a potential similarity of the signaling networks. In addition, the FMIP method

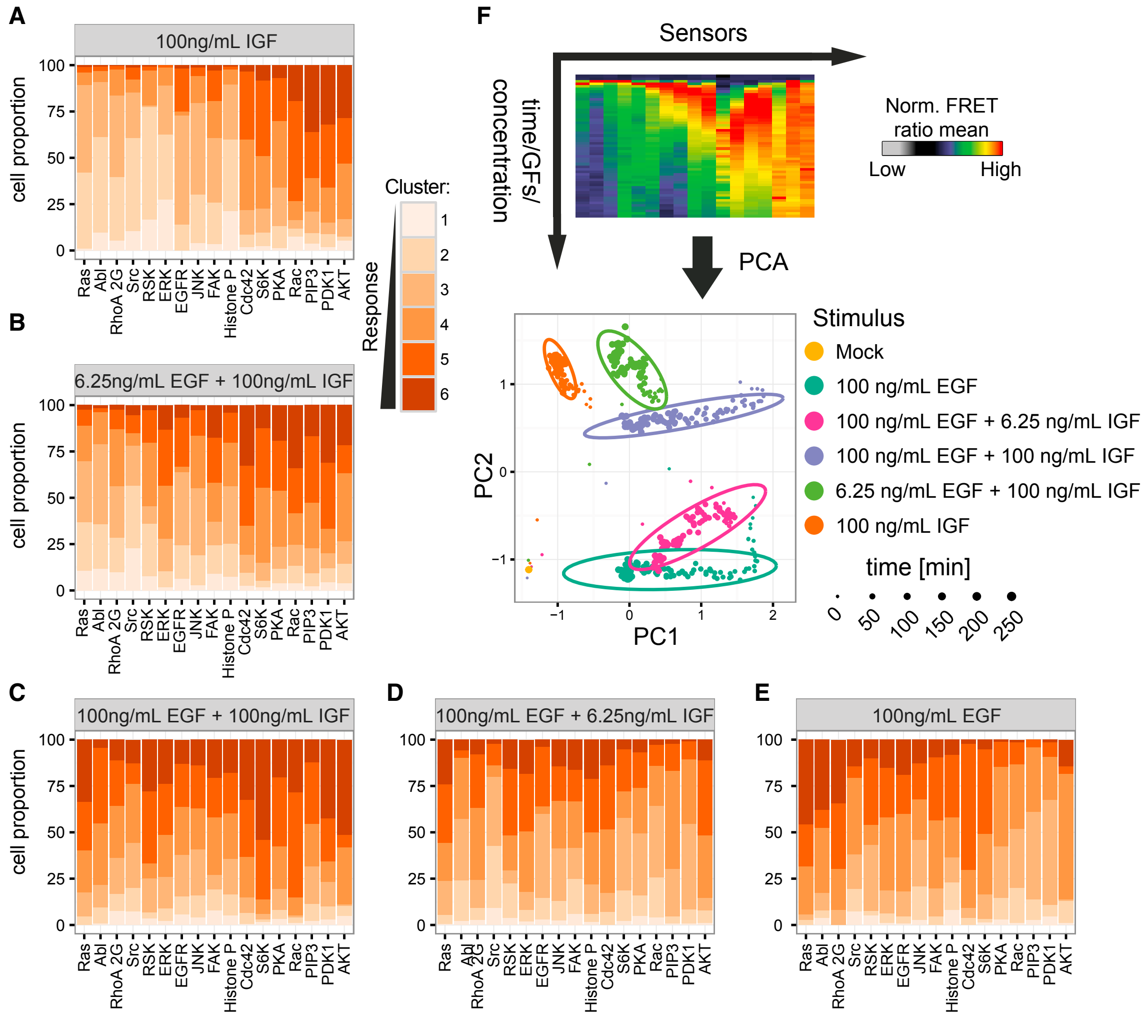

Figure 3. Crosstalk of the EGF and IGF-1 Signaling Networks

(A–E) Distribution of representative FRET biosensors time series in response to various stimuli. HeLa cells were treated with 100 ng/mL IGF-1 (A), 6.25 ng/mL EGF + 100 ng/mL IGF-1 (B), 100 ng/mL EGF + 100 ng/mL IGF-1 (C), 100 ng/mL EGF + 6.25 ng/mL IGF-1 (D), and 100 ng/mL EGF (E). n > 1,296 cells.

(F) Principal component analysis of the average response of the growth factor. Each dot represents a single time point while the ovals indicate 80% of time points of the same treatment.

coupled to a PCA analysis illustrated an ability of signaling networks to be tuned with remarkable precision and achieve a potentially unlimited number of different states depending on the relative contribution of the activating stimuli.

Finally, to obtain a mechanistic insight into the integration of EGF and IGF-1 signaling, we focused on individual signaling nodes. To visualize the crosstalk between EGF and IGF-1, we computed a synergy score for each responding FRET biosensor to form a composite "synergy map" under various concentrations (Figures 4A, 4B, and S12). We hypothesized that if the action of EGF and IGF-1 were independent, we would observe additive effects (Ss = 0, additivity), whereas interactions due to receptor cross-reactivity, downstream signal amplification/inhibition, or autocrine signaling would cause non-additivity (Ss > 0, synergism; Ss < 0, antagonism) (Figures 4A and 4B). Surprisingly the synergy map was dependent on the concentration of growth factors, which explains the non-proportional shift in the PCA space (Figure 4B). For example, the highest concentration of both EGF and IGF-1 strongly activate S6K although with different dynamics, but the combined action of the two is lower than that expected for their additive effect (antagonism) (Figure 4C). However, the decrease of either EGF or IGF-1 concentration induces the strongly or no synergistic effect in the late phase of S6K activation, respectively. Surprisingly, the proportional decrease of EGF and IGF-1 concentrations led to a slight antagonism in S6K activation. The comparison of the average synergy score reveals that under the highest concentrations of EGF and IGF-1 Ss is negative (Ss = −0.24), suggesting the saturation of the shared molecular signals of these growth factors (Figure 4B). Notably, decreased concentration of one of the growth factors or both of them in the co-treatment elevated the average synergy score (100 ng/mL EGF + 6.25 ng/mL IGF, Ss = 0.32; 6.25 ng/ml EGF + 100 ng/mL IGF, Ss = 0.27; 12.5 ng/mL EGF + 12.5 ng/mL IGF, Ss = 0.18), indicating strong

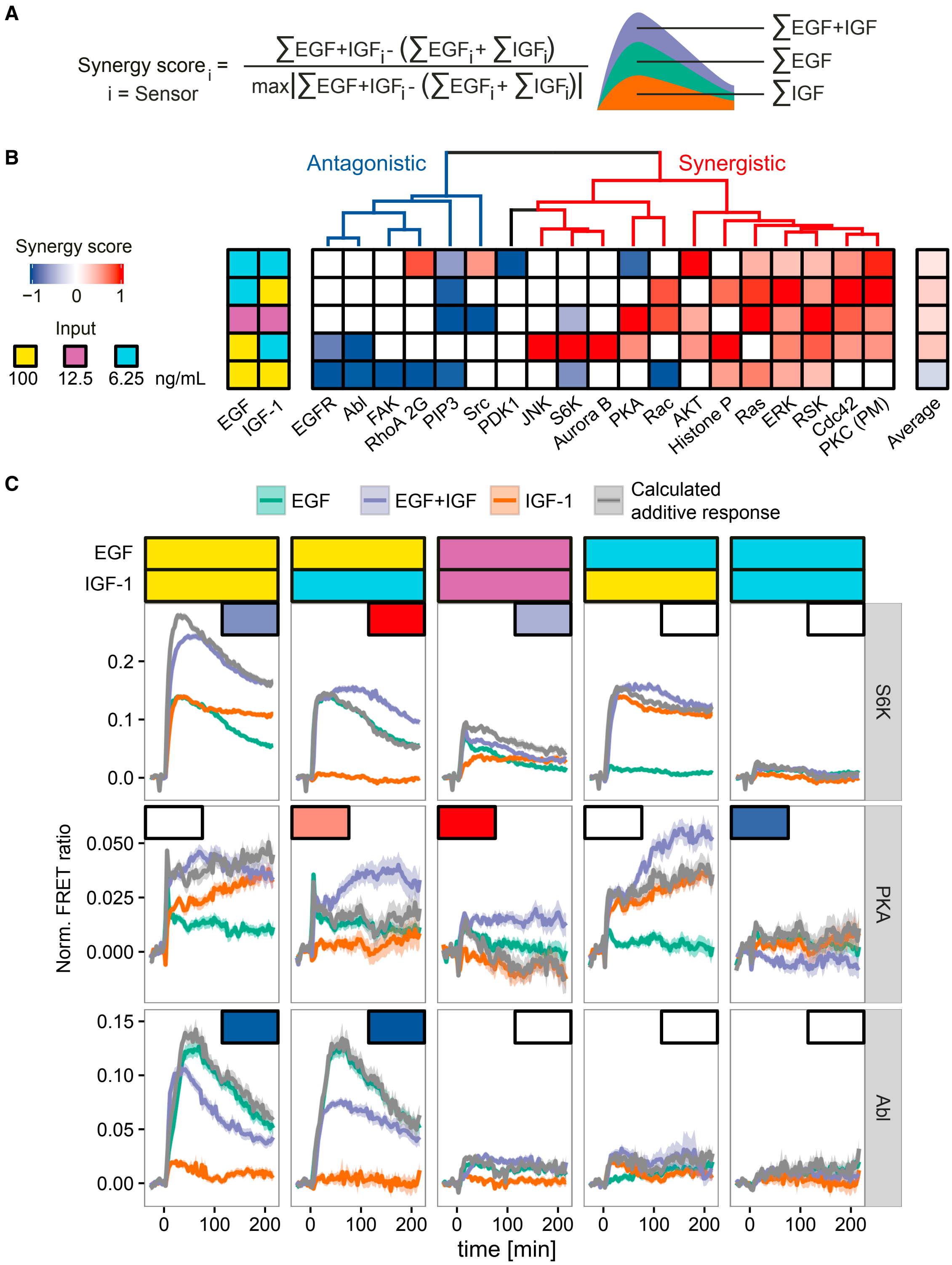

Figure 4. Crosstalk of the EGF and IGF-1 Signaling Networks

(A) Presentation of the synergy score.

(B) Synergy map which reflects antagonism, additivity, and synergy due to GFs crosstalk. HeLa cells were stimulated with 100 ng/mL EGF, 12.5 ng/mL EGF, 6.25 ng/mL EGF, 100 ng/mL IGF-1, 12.5 ng/mL IGF-1, and 6.25 ng/mL IGF-1 alone and their combination. The matrix was hierarchically clustered with the Euclidean metric and the Ward's linkage. All insignificant values ($p \geq 0.05$) are in white.

(C) Examples of two classes of signaling molecules separated by concentration-dependent synergy properties: synergistic (PKA and S6K) and antagonistic, Abl. Rectangles depict the synergy scores from (B). Data represent mean ± SEM.

synergy between EGF and IGF-1. The hierarchical clustering also indicates that the signaling activities can be clearly segregated into at least two groups by synergy properties: synergistic and antagonistic responses (Figure 4B). Taken together, these data show concentration-dependent EGF/IGF-1 crosstalk and support the concept that dynamically encoded information (Kholodenko et al., 2010; Purvis and Lahav, 2013) is a potential mechanism by which information from combined stimuli integrate to regulate signal-specific cellular processes. Thus, the FMIP provides a unique technique enabling the investigation of signaling network crosstalk at a systems level in an unbiased way.

DISCUSSION

By using microarray technology, we developed a powerful method to image FRET biosensors for monitoring enzyme activities in a high-throughput manner. The resulting platform (FMIP) is useful for simultaneously measuring a variety of intracellular signaling responses to a given set of stimuli at a single-live-cell level. We demonstrate the ability of this platform to profile perturbations caused by functional mutations of EGFR and a clinically important MEK inhibitor (AZD6244, selumetinib). We also show that this platform is able to partially resolve the signaling crosstalk between EGFR and IGF-1R.

As a way to demonstrate the power of the technique, we show that the MEK inhibitor AZD6244 is able to dramatically inhibit the activity of ERK, RSK, and, surprisingly, PKA following stimulation by EGF in HeLa cells (Figures S7A and S14). Moreover, comparing the synergy score pattern between PKA and RSK across various concentrations of EGF and IGF-1 indicated that high synergy in RSK correlates with high synergy in PKA responses in the presence of EGF (Figure 4B). We also observed that the activity of PKA strongly correlated with the activity of RSK and ERK (Figure S13) in the presence of EGF. Overall, the data suggest that the activity of RSK and PKA is co-regulated or that one of the proteins positively regulates the other. In support of this hypothesis, it was shown previously that RSK can directly bind to PKA and regulate its activity (Gao and Patel, 2009). Also, this study on B82L cells reported that active RSK attenuated the phosphorylation of the Bcl-xL/Bcl-2-associated death promoter on Ser-115 by PKA, indicating a negative regulation of PKA activity by active RSK. In contrast, our data indicate a more complex interplay between RSK and PKA. In our experiments, we observed activation of PKA by various concentrations of EGF (Figure S3), but in the study mentioned above there is no difference between EGF-treated and -untreated cells. The difference was observed only upon additional treatment with 8-pCPT-cAMP (the slowly hydrolyzing analog of cAMP) giving rise to the possibility that the experimental setup was less sensitive. We cannot rule out effects due to differences in the signaling network architecture and/or in the level of gene expression between HeLa and B82L cells, but we suspect that monitoring PKA activity over 5 hr with the AKAR3EV FRET biosensor more accurately reflects the dynamic activity of PKA under quasi-physiological conditions.

A long-standing question in the field of cellular signaling is how signaling networks integrate and process information from multiple extracellular cues. It was recently suggested that interactions between two stimuli are highly combinatorial and might be segregated into at least ten interaction modes governing gene expression (Cappuccio et al., 2015). By using similar synergy score metrics to those described previously (Lee and Diamond, 2015; Natarajan et al., 2006), we show that the interaction modes (synergy, additivity, or antagonism) between EGF and IGF-1 are highly variable among downstream signaling molecules (Figure 4B). Moreover, we demonstrate that the interaction mode is concentration dependent (Figures 4B and 4C). Thus, it will be of great interest to determine how information from other physiological stimuli including growth factors such as hepatocyte, fibroblast, or nerve growth factors is integrated by signaling networks and most importantly to understand how this combinatorial code is interpreted (or decoded) on the level of gene expression. We believe that extensive gene expression studies in combination with signaling data provided by our imaging platform will help to answer those questions in the near future.

Our FMIP platform currently uses only FRET biosensors but could be extended with any fluorescence-based biosensor (such as translocation probes) (Regot et al., 2014) or in combination with small interfering RNAs. The platform could also be adapted for hard-to-transfect primary cells by implementation of lentivirus-infected cell microarrays (Bailey et al., 2006). Importantly, the FMIP could be combined with a perfusion system and optogenetics (Toettcher et al., 2013) or caged molecules (Nadler et al., 2013) to manipulate the extracellular environment and activity of isolated molecules in a highly controlled manner, respectively. As the effectiveness of FMIP is strongly dependent on the performance of each sensor and current settings allow monitoring of up to 384 FRET biosensors in a single experiment, we believe that future advances in biosensor development to cover additional parameters and to provide improved dynamic range, sensitivity, and selectivity of existing biosensors will further improve the performance of the platform in the near future.

We anticipate that this powerful platform will be useful for understanding signal transduction mechanisms and will help to describe signaling networks in a more systematic way. We expect applications of the FMIP to study drug action, to determine side effects of drugs on vital cellular functions, and to understand cellular mechanisms of therapeutic resistance to drug treatments after long-term exposure.

SIGNIFICANCE

To better understand complex intracellular signaling networks, technologies that enable simultaneous measurement of protein activities and second messenger concentrations are essential for basic and clinical research. FRET biosensors are able to visualize the dynamic *activity* of proteins, not merely abundance, and concentrations of second messengers with high temporal resolution in a single live cell. However, so far the high-content capability was limited to a few FRET biosensors. Here, by using cell microarray technology in combination with FRET biosensors and live-cell imaging, we developed a high-content imaging platform that allows visualizing of hundreds of FRET biosensors in a single experiment. As a proof of principle, by employing 40 FRET biosensors, we have demonstrated the capability of this platform to monitor perturbations in the EGF signaling network caused by overexpression of constitutively active

EGFR and by a clinically relevant MEK inhibitor. We also show that our platform is able to resolve a crosstalk between growth factors. Our technique provides the potential to study signaling networks in disease and hence improve drug discovery, to identify mechanism of action and side effects of therapeutic candidates, as well as to investigate principle signal transduction mechanisms.

EXPERIMENTAL PROCEDURES

Cells and Reagents

HeLa Kyoto cells were a kind gift of R. Pepperkok (European Molecular Biology Laboratory, Germany). HeLa Kyoto cells were maintained in low glucose DMEM (Life Technologies) supplemented with 10% fetal bovine serum (FBS) and with 100 μg/mL of Primocin (InvivoGen). H838 cells were maintained in high glucose DMEM (Life Technologies) supplemented with 10% FBS and with 100 μg/mL of Primocin (InvivoGen). Starvation media for HeLa Kyoto cells contained low glucose DMEM (Life Technologies) and 100 μg/mL of Primocin (InvivoGen). Starvation of H838 cells and H838 cells expressing EGFR with both activating L858R and resistant T790M mutations was performed in high glucose DMEM (Lonza) supplemented with 1 mg/mL of BSA (Sigma), 2 mM L-glutamine (Invitrogen), 100 units/mL penicillin, and 100 g/mL streptomycin (Pen-Strep, Invitrogen). AZD6244 was purchased from Selleck Chemicals (selumetinib). EGF and IGF-1 were obtained from Sigma.

Establishment of a Stable Cell Line

cDNA of the human EGFR harboring the L858R and T790M mutation was purified from H1975 cells (ATCC) by extraction of mRNA with the RNeasy Kit (QIAGEN) and reverse transcription (Agilent). The cDNA was used as template for PCR with gene-specific primers containing XhoI and PacI restriction sites and subcloned into pMOWS vector containing puromycin resistance (Schilling et al., 2009). The plasmid was transiently transfected in phoenix-ampho packaging cells (Swift et al., 2001) by calcium phosphate precipitation. After 16 hr incubation, the retroviral particles were harvested from the supernatant, filtered with a 0.45 μm filter (Millipore), and used for transduction of the H838 cell line (ATCC). H838 cells were centrifuged in a 6-well plate with 1 mL viral supernatant, containing 8 mg/mL polybrene, for 3 hr at 340 × *g*. The cells were selected the following day using 1.5 μg/mL puromycin (InvivoGen, catalog no. ant-pr-1). H838-EGFR L858R/T790M cells were cultivated in DMEM medium containing 10% fetal calf serum, 1% penicillin/streptomycin, and 1.5 μg/mL puromycin.

Contact Printing

The reverse-transection approach developed in this work is a modified version of previously described protocols (Erfle et al., 2007; Piljic et al., 2011) to achieve better transfection efficiency. In brief, plasmids for reverse transfection were isolated from *Escherichia coli* using a maxi QIAfilter Plasmid Maxi Kit and diluted to concentration of 1 mg/mL. To prepare the transfection mixture, 9 μL of a 0.4 M sucrose solution in DMEM, 9 μL of DNA and 33 μL of Lipofectamine 2000 were mixed in a 96-well plate. After 20 min incubation at room temperature, 21.75 μL of solution of 0.29% gelatin in water was added to the mixture, and 24 μL of the transfection cocktail was distributed in 384-well plates. Subsequently, a plate was centrifuged briefly up to 54 × *g* at room temperature to straighten the surface of the samples and placed immediately in the contact printer. Before printing, Lab-Tek dishes were washed with 70% ethanol to increase the hydrophobicity of the Lab-Tek surface and, accordingly, to improve the shape of the spots. One-well Lab-Tek dishes were printed with a "ChipWriter" contact printer equipped with solid pins. Using PTS 600 pins, the diameter of printed spots was about 400 μm and the spot-to-spot distance was 1.125 μm. Printed 1-well Lab-Tek dishes were stored at room temperature in a gel-drying box in the presence of drying pearls.

Imaging

Cells (650,000) were seeded onto a printed glass coverslip 1-well Lab-Tek chamber. After maintaining cells in an incubator for 24–48 hr, the media were changed to starvation media (see above) at least 12–17 hr prior to imaging. To assist cellular segmentation cell lines were incubated with 7.5 nM DRAQ5 (Cell Signaling Technology). During imaging, cells were maintained in imaging medium (minimum essential medium supplemented with 100 U/mL of penicillin, 100 μg/mL of streptomycin, and 30 mM of HEPES) at 37°C without CO_2. Time-lapse imaging was performed on an Olympus IX83 microscope equipped with a Hamamatsu ImagEM CCD camera and an environmental chamber using 20× 0.70 numerical aperture (NA) or 10× 0.40 NA and 436/20 excitation filter, a CFP/yellow fluorescent protein (YFP) dual-band beam splitter (51017bs; Chroma), and two emission filters (470/30 for CFP and 535/50 for YFP) that were controlled by a filter wheel. The images were captured with xCELLence software at 3 min interval.

Imaging Data Analysis

Primarily, the binary mask for the nucleus was defined using a DRAQ5 marker and all channels were combined in a single file by the in-house developed ImageJ Macro (Schindelin et al., 2012). Subsequently, images were analyzed with ImageJ Macro FluoQ as depicted in Figure S1 and as described previously. In brief, the mean of the thresholded background calculated with a histogram-based "Triangle" algorithm is subtracted from each pixel. Images were then smoothed with a median filter (radius size = 2) and transformed to a 32-bit float. To segment cells, a binary mask was created by Huang's fuzzy thresholding method. The signal intensity that was equal or close to intensity of the background was set as NaN value due to Huang's fuzzy thresholding. The CFP intensity of cells ranged from 700 to 8,000 a.u. depending on the FRET biosensor. This image analysis pipeline automatically excludes low-expressing cells and avoids erroneous FRET ratios. The FluoQ macro identified cell nuclei from the images of a binary mask. The cell outlines were then identified using the watershed algorithm. Finally, the particle analyzer plug-in was used to define regions of interest (ROIs). To simplify and fasten the image analysis pipeline that is capable of analyzing 13,900 images from a single experiment in a reasonable time window we averaged the intensity of FRET, CFP, and FRET ratios over each ROI. Taken into account that many signaling pathways are compartmentalized, the spatial variation of the sampled signals and significant fluctuations might be averaged out due to averaging over each ROI. In all experiments, the FRET ratio from before stimulation was averaged and used for normalization. FluoQ provided the mean pixel intensity of each ROI over time, saved all measured data, and calculated parameters in a text file format (EXPNAME.txt).

Statistical Analysis

To perform data analysis, a text file was loaded into the program R (R Development Core Team, 2012). The R package "ggplot2" (Wickham, 2009) was used to visualize the data. Clustering was performed using the heatmap.2 function of the R package "gplots" (Warnes et al., 2012). We observed that the intensity of CFP and FRET channels is increasing over time most probably due to a constant increase in FRET biosensor expression, as depicted in Figure S6A, taking into account that we performed the imaging over 5 hr. Although the intensity of CFP and FRET channels is increased the FRET ratio is much more stable (Figure S6A). Therefore, to minimize the impact of FRET sensor expression and/or photobleaching, the single-cell time courses were normalized to the FRET ratio mean of cells treated with a vehicle (imaging medium) at each time point and to the average of data points prior to the stimulation in Figures 1B, 1C, 3A–3E, 4B, 4C, S3A, S3B, S9, S10, and S11–S13. If not stated otherwise, the overall FRET ratio for a reporter is represented as a normalized FRET ratio mean of all individual cells from identical conditions ± SEM, where the SEM is calculated from all cells in all experiments under identical conditions. In Figures 1B, 1C, and S4B, the maximum observed FRET ratio value of each biosensor was additionally used for normalization.

The statistical significance (p value) for Kendall's tau correlating coefficients was determined using the program R. The exact p value is computed if there are less than 50 paired samples containing finite values and there are no ties. Otherwise, the test statistic is the estimate scaled to zero mean and unit variance, and is approximately normally distributed. In Figure 2C, PCA was performed on a 340 × 14 matrix, with 85 time points, and 4 cell lines each with 14 FRET biosensors. In Figure 3F, PCA was performed on a 546 × 17 matrix, with 546 time points, growth factors, and dose each with 17 FRET biosensors. We used the prcomp function with centering and scaling of the program R to perform PCA analysis and the R package "ggplot2" for visualization.

To estimate the additive effects of a combined EGF and IGF treatment, we first normalized every single-cell time-trace by dividing each time point with the average of data points prior to the stimulation and with the mean value of untreated cells followed by a subtraction of 1. Then we computed the area under the curve for each individual cell. The data were analyzed for normality using the Shapiro-Wilk test and the quantile-quantile plot. We then averaged the area under the curve for the corresponding sensor and stimulus and computed the SEM to estimate the error for calculated additive response. We calculated the expected response by simple addition of the mean of the area under the curve of "EGF only" and "IGF-1 only" treatments. To estimate the combined error, we performed a simple error propagation of the individual SEs of the mean using a variance formula (Ku, 1966). Student's two-sample t test was used to determine whether there was a statistically significant difference between the means of experimental and the expected (calculated) additivity. The p values obtained were corrected for multiple testing (Benjamini and Hochberg, 1995). The synergy score were calculated as the difference between the means of experimental and expected (calculated) additivity. In Figure 4B, the synergy scores with all insignificant difference ($p \geq 0.05$) are in white (Figure S12). To simplify visualization, the synergy score was scaled to the maximum synergy score observed for a FRET biosensor, giving a value that ranges from −1 (antagonism) to +1 (synergy).

SUPPLEMENTAL INFORMATION

Supplemental Information includes fourteen figures and one table and can be found with this article online at http://dx.doi.org/10.1016/j.chembiol.2016.11.008.

AUTHOR CONTRIBUTIONS

C.S., V.L., and D.K. devised the method. V.L. and D.K. performed the initial implementation. D.K. designed, performed, and analyzed the experiments. D.K. and F.St. performed image and data analysis. F.Sa. and U.K. generated cell lines. D.K., V.L., and C.S. wrote the paper. All authors reviewed the manuscript.

ACKNOWLEDGMENTS

We thank R. Pepperkok and M. Schultz for critical reading of the manuscript and members of the Schultz laboratory for helpful discussion. We are grateful to the ALMF team at EMBL for excellent technical support and B. Neumann for help with microarray spotting. We thank M. Matsuda, M.C. Parrini, Y. Ohba, J. Zhang, O. Pertz, Y. Wang, A.Y. Ting, J Pines, A.C. Newton, O. Griesbeck, T.M. Kapoor, S. Vogel, M.A. Lampson, and M. Yoshida for providing FRET biosensors. The work was supported by EMBL, the German Federal Ministry of Education and Research (LungSys II), the DFG (SFB 1129) and the Joachim Herz Foundation. The work in the Klingmüller group was supported by the German Federal Ministry of Education and Research (BMBF) through the LungSysII consortium and the German Center for Lung Research (DZL).

Received: May 6, 2016
Revised: September 19, 2016
Accepted: November 14, 2016
Published: December 8, 2016

REFERENCES

Bailey, S.N., Ali, S.M., Carpenter, A.E., Higgins, C.O., and Sabatini, D.M. (2006). Microarrays of lentiviruses for gene function screens in immortalized and primary cells. Nat. Methods *3*, 117–122.

Bakal, C., Linding, R., Llense, F., Heffern, E., Martin-Blanco, E., Pawson, T., and Perrimon, N. (2008). Phosphorylation networks regulating JNK activity in diverse genetic backgrounds. Science *322*, 453–456.

Bekaii-Saab, T., Phelps, M.A., Li, X., Saji, M., Goff, L., Kauh, J.S., O'Neil, B.H., Balsom, S., Balint, C., Liersemann, R., et al. (2011). Multi-institutional phase II study of selumetinib in patients with metastatic biliary cancers. J. Clin. Oncol. *29*, 2357–2363.

Bendall, S.C., Nolan, G.P., Roederer, M., and Chattopadhyay, P.K. (2012). A deep profiler's guide to cytometry. Trends Immunol. *33*, 323–332.

Benjamini, Y., and Hochberg, Y. (1995). Controlling the false discovery rate: a practical and powerful approach to multiple testing. J. R. Stat. Soc. *57*, 289–300.

Blagoev, B., Ong, S.E., Kratchmarova, I., and Mann, M. (2004). Temporal analysis of phosphotyrosine-dependent signaling networks by quantitative proteomics. Nat. Biotechnol. *22*, 1139–1145.

Borisov, N., Aksamitiene, E., Kiyatkin, A., Legewie, S., Berkhout, J., Maiwald, T., Kaimachnikov, N.P., Timmer, J., Hoek, J.B., and Kholodenko, B.N. (2009). Systems-level interactions between insulin-EGF networks amplify mitogenic signaling. Mol. Syst. Biol. *5*, 256.

Cappuccio, A., Zollinger, R., Schenk, M., Walczak, A., Servant, N., Barillot, E., Hupe, P., Modlin, R.L., and Soumelis, V. (2015). Combinatorial code governing cellular responses to complex stimuli. Nat. Commun. *6*, 6847.

Cheong, R., Wang, C.J., and Levchenko, A. (2009). High content cell screening in a microfluidic device. Mol. Cell. Proteomics *8*, 433–442.

de la Cruz-Herrera, C.F., Campagna, M., Lang, V., Del Carmen Gonzalez-Santamaria, J., Marcos-Villar, L., Rodriguez, M.S., Vidal, A., Collado, M., and Rivas, C. (2015). SUMOylation regulates AKT1 activity. Oncogene *34*, 1442–1450.

Erfle, H., Neumann, B., Liebel, U., Rogers, P., Held, M., Walter, T., Ellenberg, J., and Pepperkok, R. (2007). Reverse transfection on cell arrays for high content screening microscopy. Nat. Protoc. *2*, 392–399.

Fujita, Y., Komatsu, N., Matsuda, M., and Aoki, K. (2014). Fluorescence resonance energy transfer based quantitative analysis of feedforward and feedback loops in epidermal growth factor receptor signaling and the sensitivity to molecular targeting drugs. FEBS J. *281*, 3177–3192.

Gao, X., and Patel, T.B. (2009). Regulation of protein kinase A activity by p90 ribosomal S6 kinase 1. J. Biol. Chem. *284*, 33070–33078.

Giesen, C., Wang, H.A., Schapiro, D., Zivanovic, N., Jacobs, A., Hattendorf, B., Schuffler, P.J., Grolimund, D., Buhmann, J.M., Brandt, S., et al. (2014). Highly multiplexed imaging of tumor tissues with subcellular resolution by mass cytometry. Nat. Methods *11*, 417–422.

Grecco, H.E., Roda-Navarro, P., Girod, A., Hou, J., Frahm, T., Truxius, D.C., Pepperkok, R., Squire, A., and Bastiaens, P.I. (2010). In situ analysis of tyrosine phosphorylation networks by FLIM on cell arrays. Nat. Methods *7*, 467–472.

Hughes, A.J., Spelke, D.P., Xu, Z., Kang, C.C., Schaffer, D.V., and Herr, A.E. (2014). Single-cell western blotting. Nat. Methods *11*, 749–755.

Kholodenko, B.N., Hancock, J.F., and Kolch, W. (2010). Signalling ballet in space and time. Nature reviews. Mol. Cell. Biol. *11*, 414–426.

Ku, H.H. (1966). Notes on the use of propagation of error formulas. J. Res. Natl. Bur. Stand. *70C*, 262.

Kubota, H., Noguchi, R., Toyoshima, Y., Ozaki, Y., Uda, S., Watanabe, K., Ogawa, W., and Kuroda, S. (2012). Temporal coding of insulin action through multiplexing of the AKT pathway. Mol. Cell *46*, 820–832.

Lee, M.Y., and Diamond, S.L. (2015). A human platelet calcium calculator trained by pairwise agonist scanning. PLoS Comput. Biol. *11*, e1004118.

Lemmon, M.A., and Schlessinger, J. (2010). Cell signaling by receptor tyrosine kinases. Cell *141*, 1117–1134.

Liu, P., Begley, M., Michowski, W., Inuzuka, H., Ginzberg, M., Gao, D., Tsou, P., Gan, W., Papa, A., Kim, B.M., et al. (2014). Cell-cycle-regulated activation of Akt kinase by phosphorylation at its carboxyl terminus. Nature *508*, 541–545.

Miyawaki, A. (2003). Visualization of the spatial and temporal dynamics of intracellular signaling. Dev. Cell *4*, 295–305.

Nadler, A., Reither, G., Feng, S., Stein, F., Reither, S., Muller, R., and Schultz, C. (2013). The fatty acid composition of diacylglycerols determines local signaling patterns. Angew Chem. Int. Ed. Engl. *52*, 6330–6334.

Natarajan, M., Lin, K.M., Hsueh, R.C., Sternweis, P.C., and Ranganathan, R. (2006). A global analysis of cross-talk in a mammalian cellular signalling network. Nat. Cell Biol. *8*, 571–580.

Newman, R.H., Fosbrink, M.D., and Zhang, J. (2011). Genetically encodable fluorescent biosensors for tracking signaling dynamics in living cells. Chem. Rev. *111*, 3614–3666.

Novakofski, K., Boehm, A., and Fortier, L. (2009). The small GTPase Rho mediates articular chondrocyte phenotype and morphology in response to interleukin-1alpha and insulin-like growth factor-I. J. Orthop. Res. *27*, 58–64.

Pawson, T. (2004). Specificity in signal transduction: from phosphotyrosine-SH2 domain interactions to complex cellular systems. Cell *116*, 191–203.

Piljic, A., de Diego, I., Wilmanns, M., and Schultz, C. (2011). Rapid development of genetically encoded FRET reporters. ACS Chem. Biol. *6*, 685–691.

Purvis, J.E., and Lahav, G. (2013). Encoding and decoding cellular information through signaling dynamics. Cell *152*, 945–956.

Regot, S., Hughey, J.J., Bajar, B.T., Carrasco, S., and Covert, M.W. (2014). High-sensitivity measurements of multiple kinase activities in live single cells. Cell *157*, 1724–1734.

Schilling, M., Maiwald, T., Hengl, S., Winter, D., Kreutz, C., Kolch, W., Lehmann, W.D., Timmer, J., and Klingmuller, U. (2009). Theoretical and experimental analysis links isoform-specific ERK signalling to cell fate decisions. Mol. Syst. Biol. *5*, 334.

Schindelin, J., Arganda-Carreras, I., Frise, E., Kaynig, V., Longair, M., Pietzsch, T., Preibisch, S., Rueden, C., Saalfeld, S., Schmid, B., et al. (2012). Fiji: an open-source platform for biological-image analysis. Nat. Methods *9*, 676–682.

Stadler, C., Rexhepaj, E., Singan, V.R., Murphy, R.F., Pepperkok, R., Uhlen, M., Simpson, J.C., and Lundberg, E. (2013). Immunofluorescence and fluorescent-protein tagging show high correlation for protein localization in mammalian cells. Nat. Methods *10*, 315–323.

Swift, S., Lorens, J., Achacoso, P., and Nolan, G.P. (2001). Rapid production of retroviruses for efficient gene delivery to mammalian cells using 293T cell-based systems. Curr. Protoc. Immunol. *Chapter 10*. Unit 10.17C.

R Development Core Team. (2012). R: A Language and Environment for Statistical Computing (R Foundation for Statistical Computing).

Thestrup, T., Litzlbauer, J., Bartholomaus, I., Mues, M., Russo, L., Dana, H., Kovalchuk, Y., Liang, Y., Kalamakis, G., Laukat, Y., et al. (2014). Optimized ratiometric calcium sensors for functional in vivo imaging of neurons and T lymphocytes. Nat. Methods *11*, 175–182.

Toettcher, J.E., Weiner, O.D., and Lim, W.A. (2013). Using optogenetics to interrogate the dynamic control of signal transmission by the Ras/Erk module. Cell *155*, 1422–1434.

Wagner, J.P., Wolf-Yadlin, A., Sevecka, M., Grenier, J.K., Root, D.E., Lauffenburger, D.A., and MacBeath, G. (2013). Receptor tyrosine kinases fall into distinct classes based on their inferred signaling networks. Sci. Signal. *6*, ra58.

Warnes, G., Bolker, B., Bonebakker, L., Gentleman, R., and Huber, W. (2012). Package ‘gplots’. http://cran.r-project.org.

Wickham, H. (2009). ggplot2: Elegant Graphics for Data Analysis (Springer).

Worster, D.T., Schmelzle, T., Solimini, N.L., Lightcap, E.S., Millard, B., Mills, G.B., Brugge, J.S., and Albeck, J.G. (2012). Akt and ERK control the proliferative response of mammary epithelial cells to the growth factors IGF-1 and EGF through the cell cycle inhibitor p57Kip2. Sci. Signal. *5*, ra19.

Ziauddin, J., and Sabatini, D.M. (2001). Microarrays of cells expressing defined cDNAs. Nature *411*, 107–110.

Cell Chemical Biology, Volume *23*

Supplemental Information

High-Content Imaging Platform for Profiling Intracellular Signaling Network Activity in Living Cells

Dmitry Kuchenov, Vibor Laketa, Frank Stein, Florian Salopiata, Ursula Klingmüller, and Carsten Schultz

Supplementary Figure 1

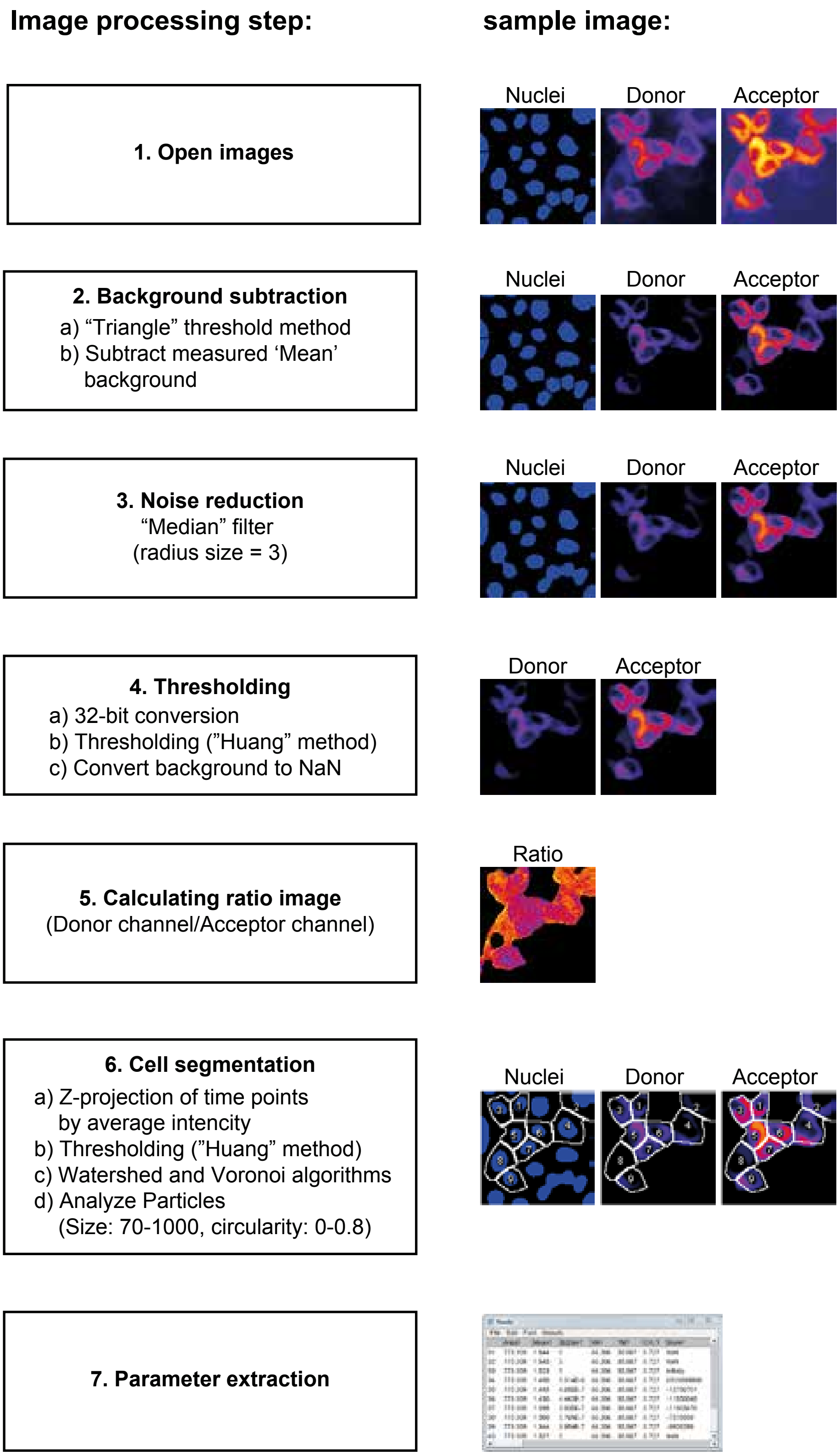

Supplementary Figure 2

A

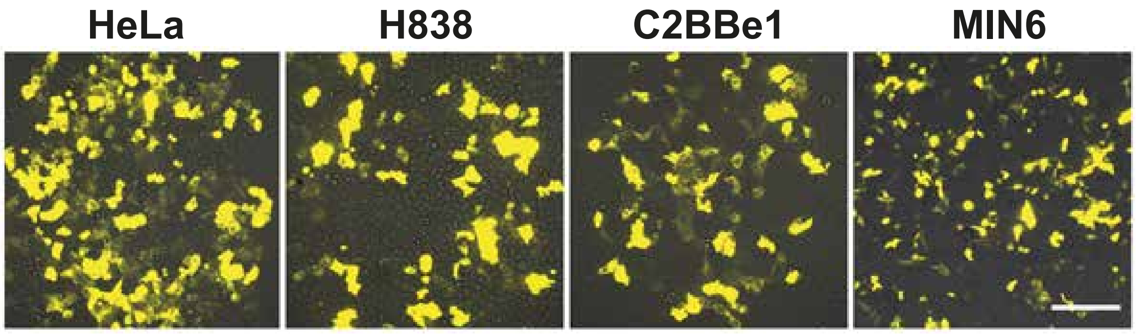

B

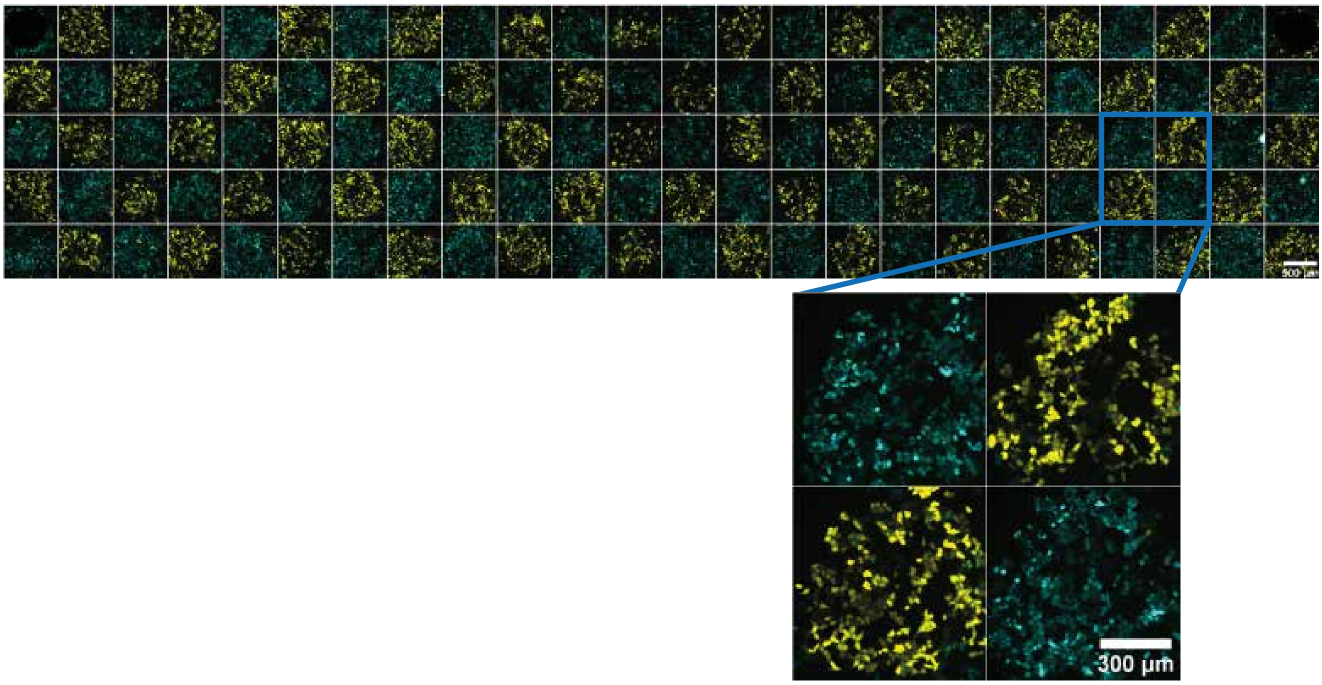

C

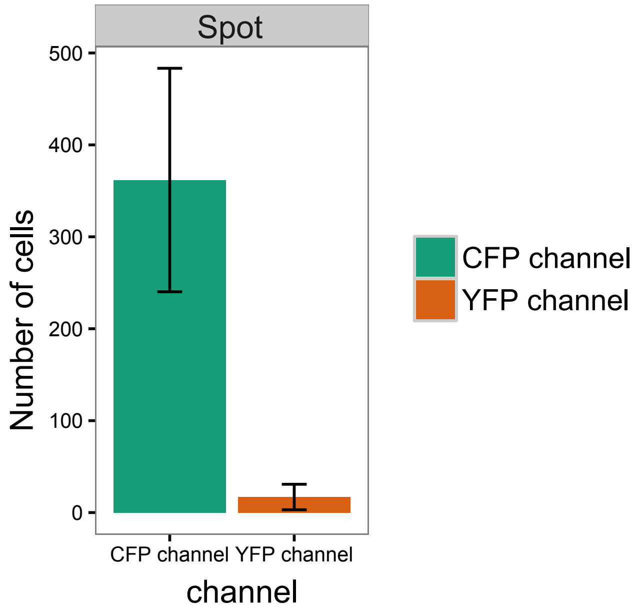

Supplementary Figure 3

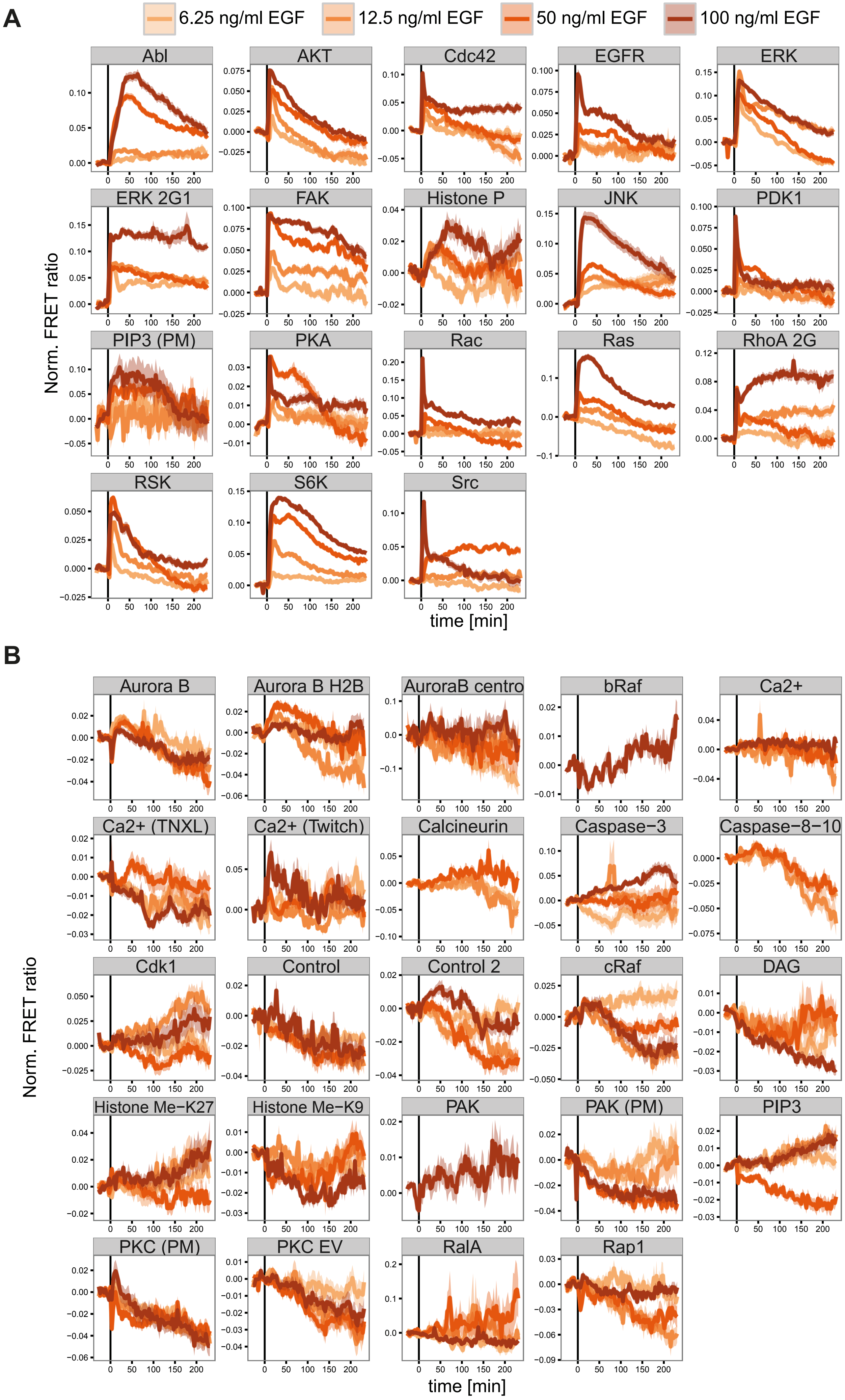

Supplementary Figure 4

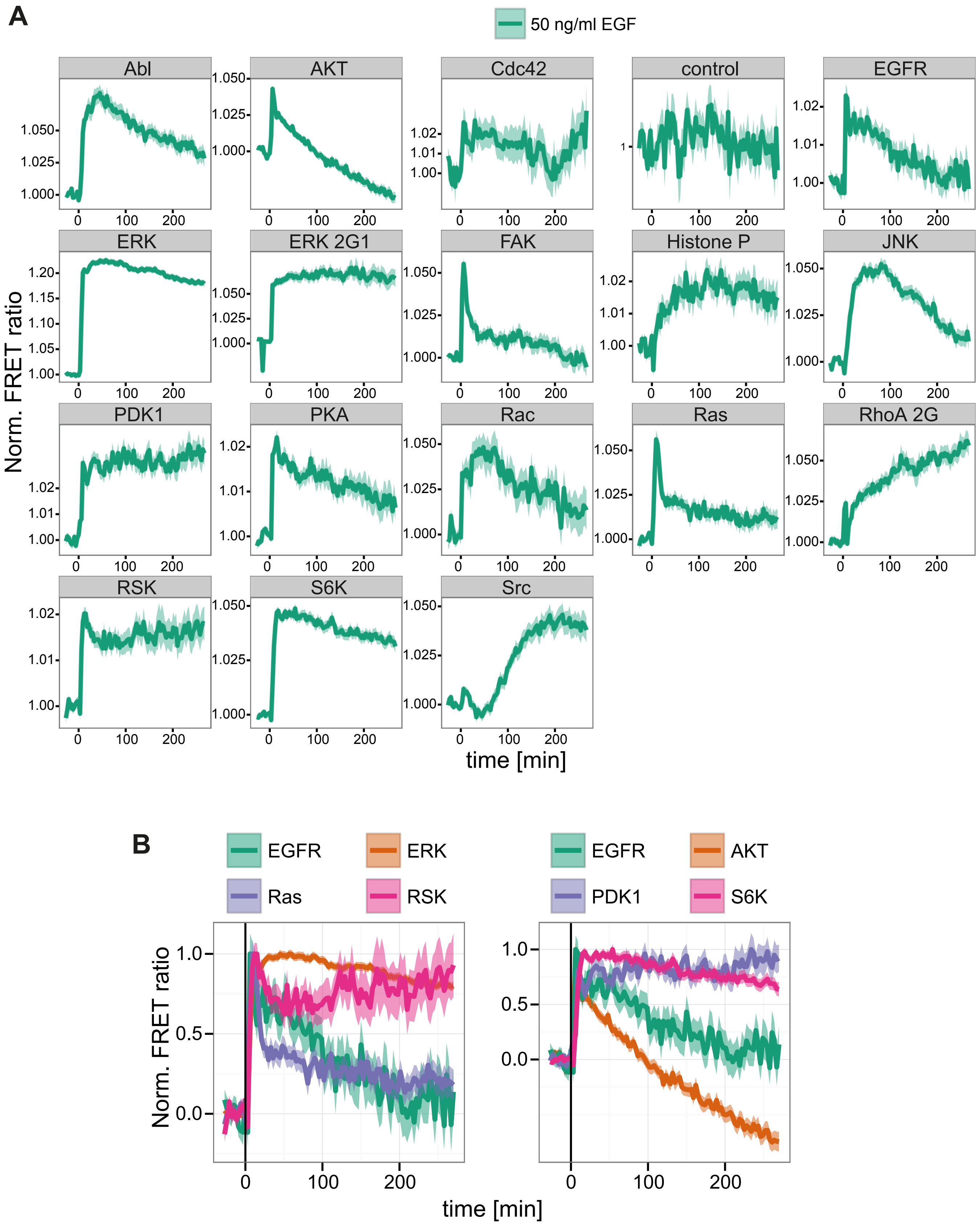

Supplementary Figure 5

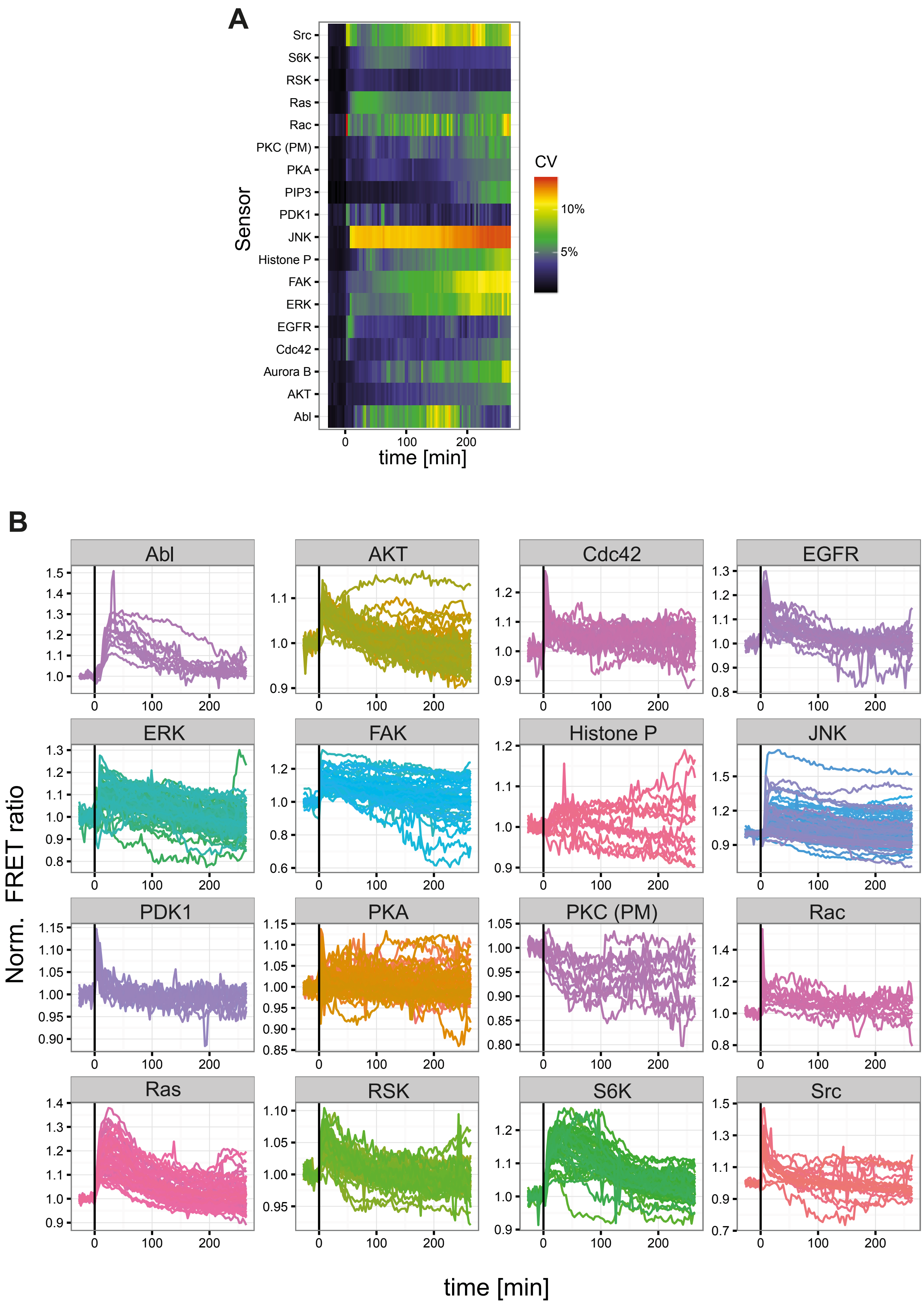

Supplementary Figure 6

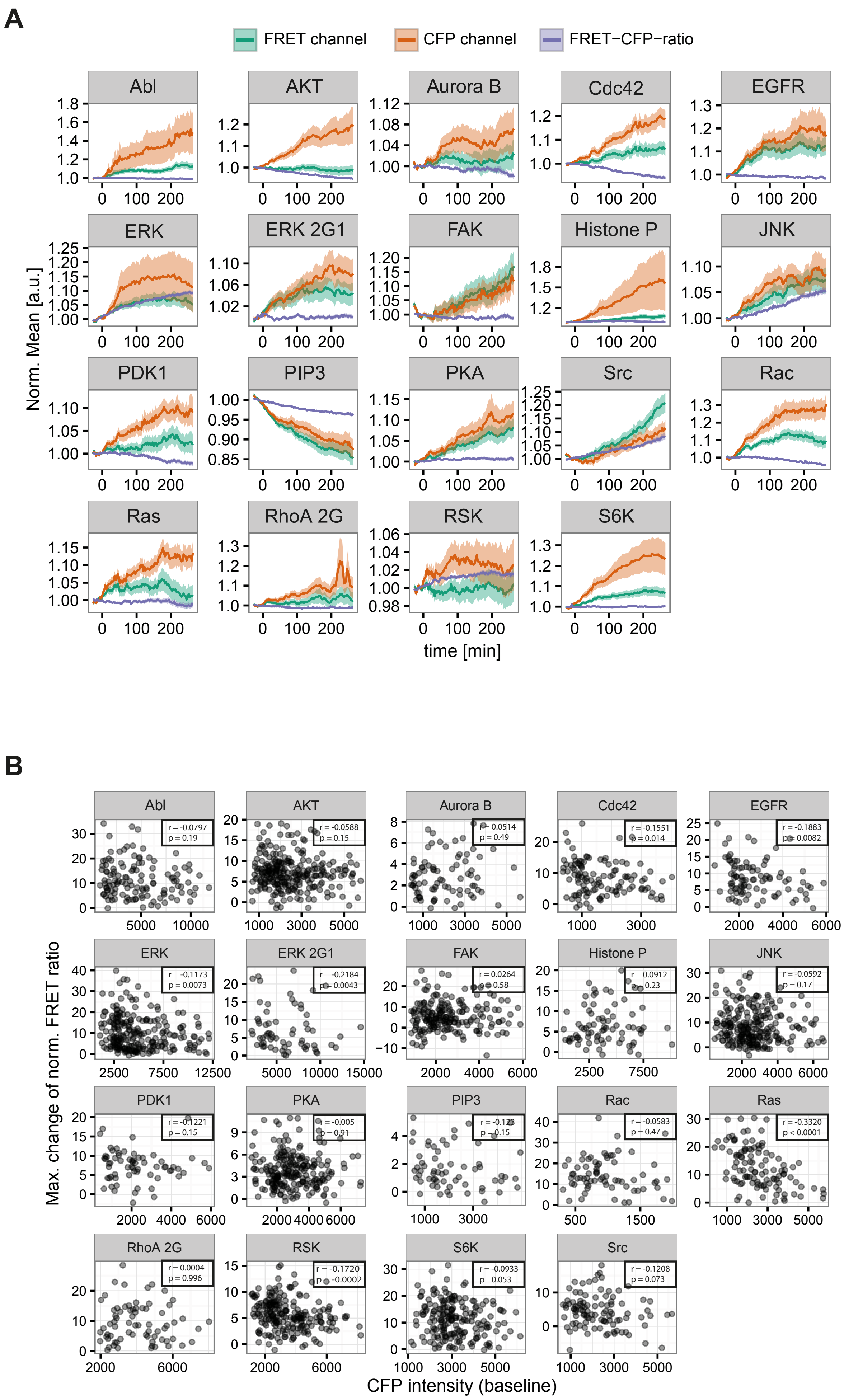

Supplementary Figure 7

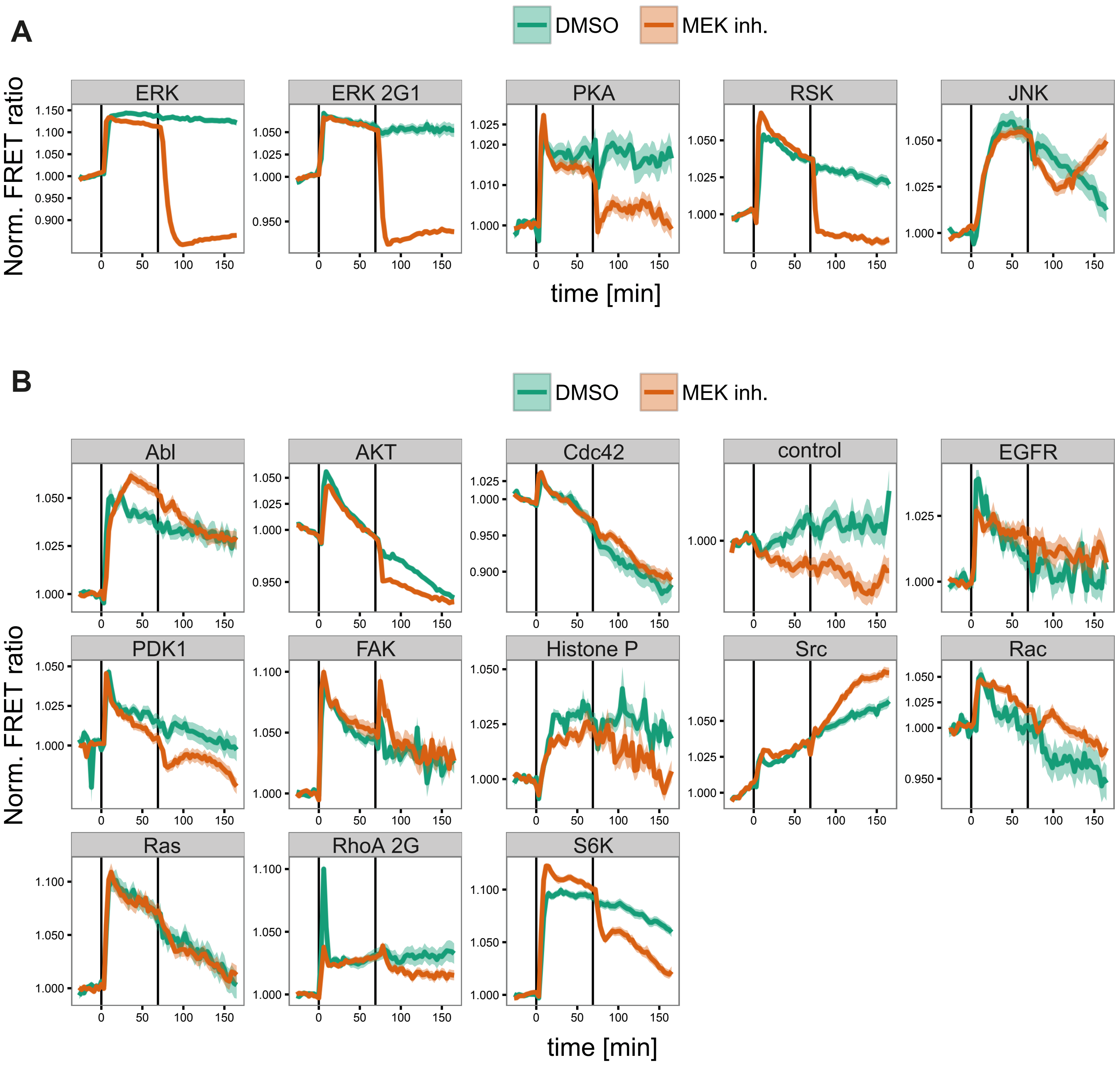

Supplementary Figure 8

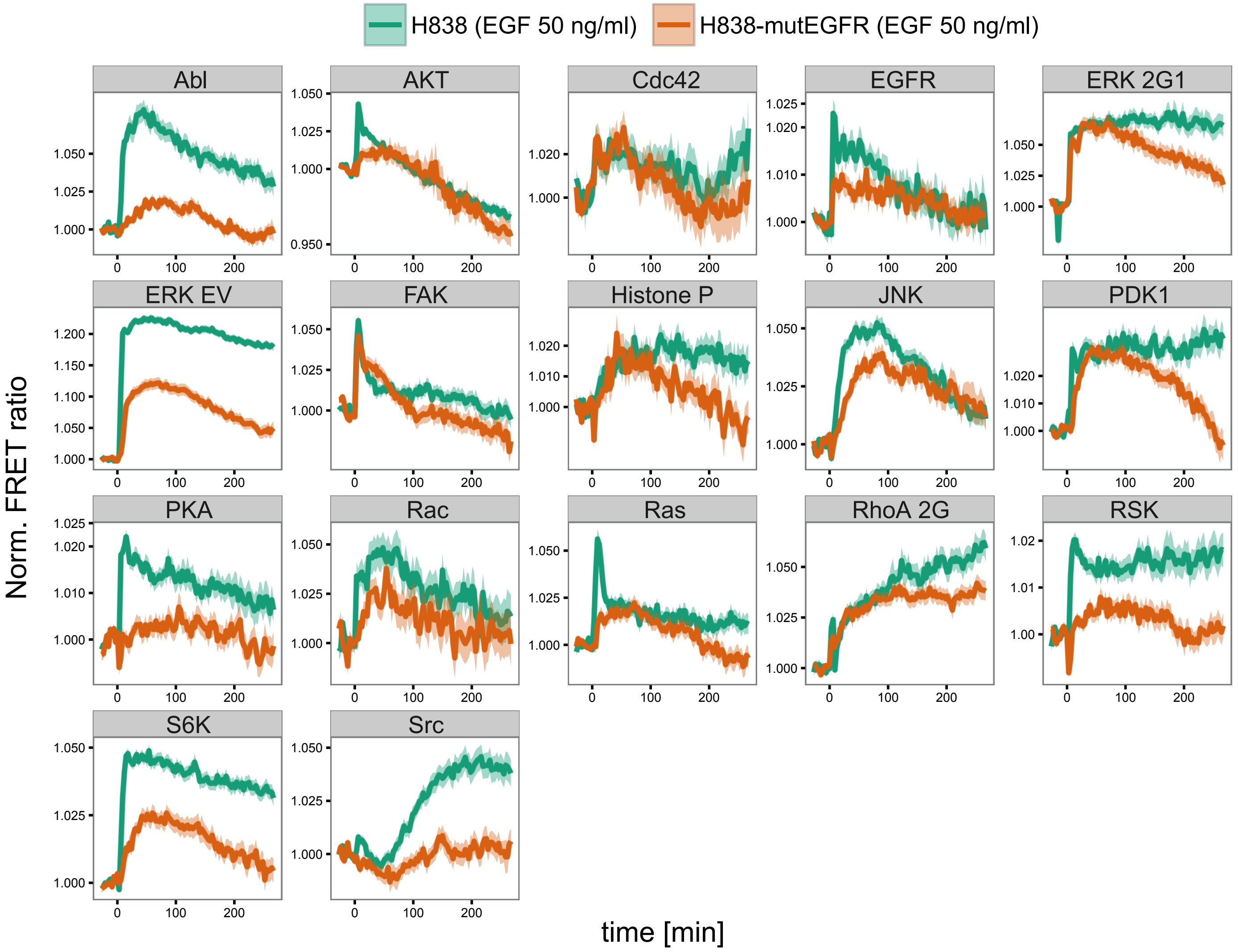

Supplementary Figure 9

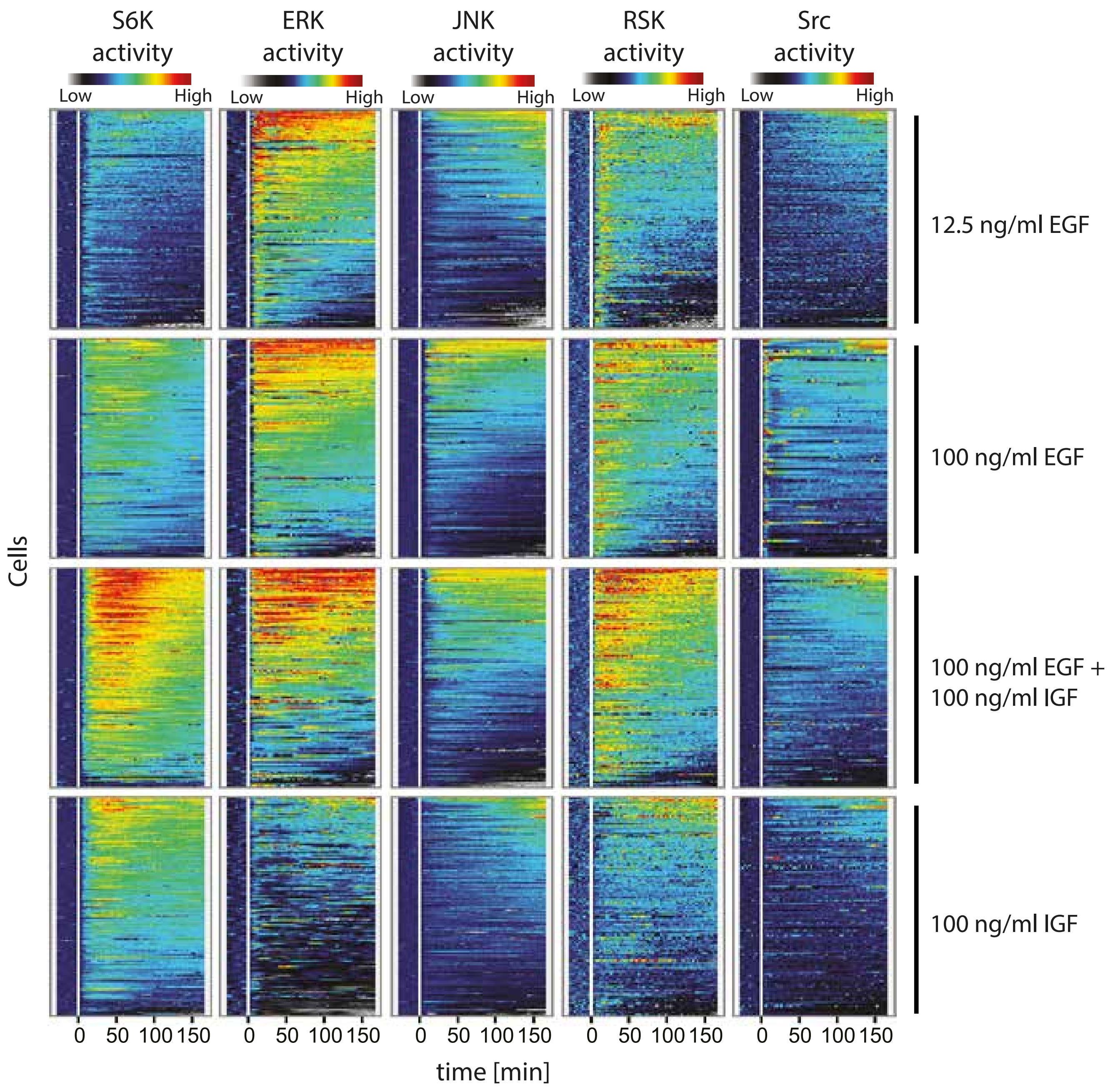

Supplementary Figure 10

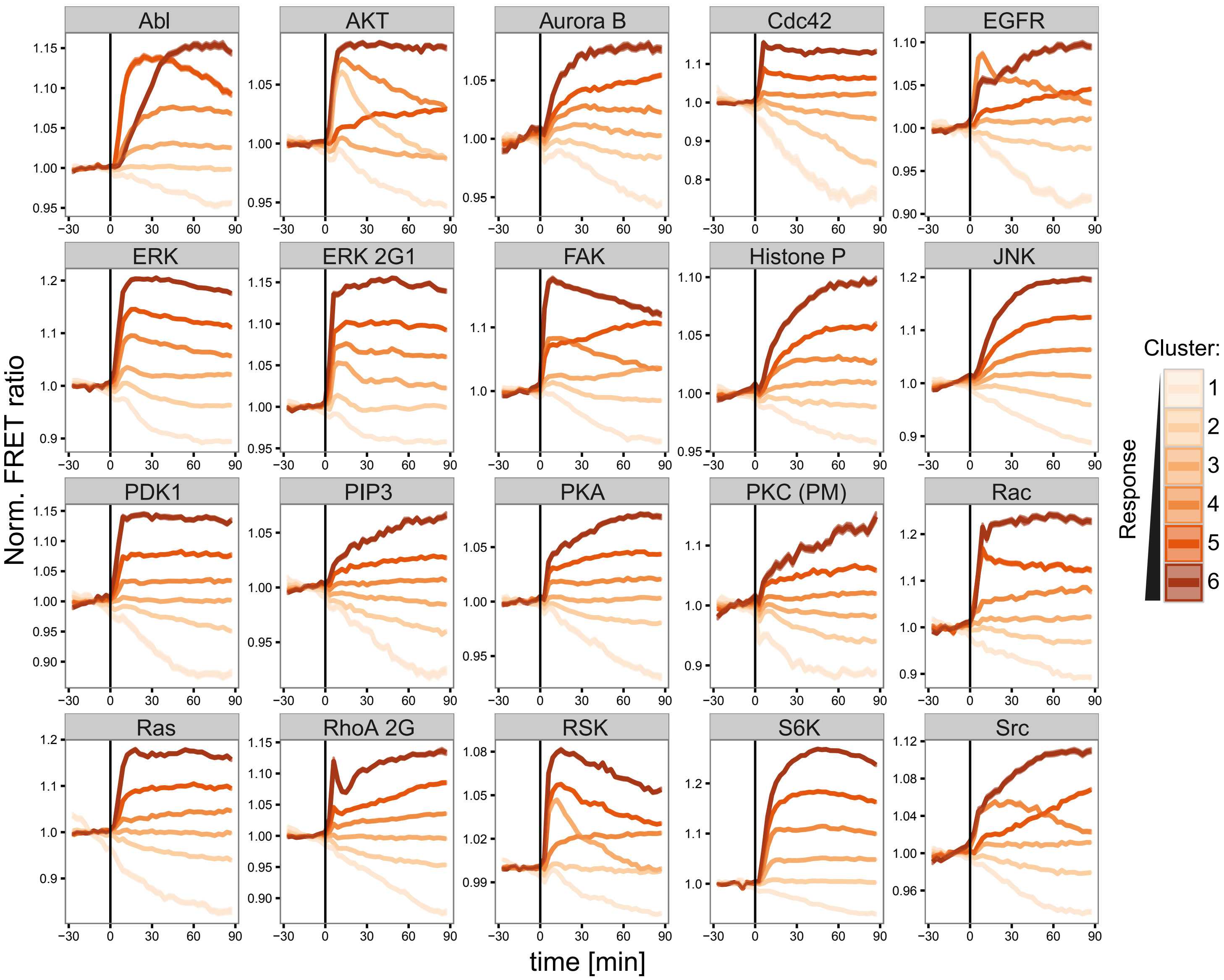

Supplementary Figure 11

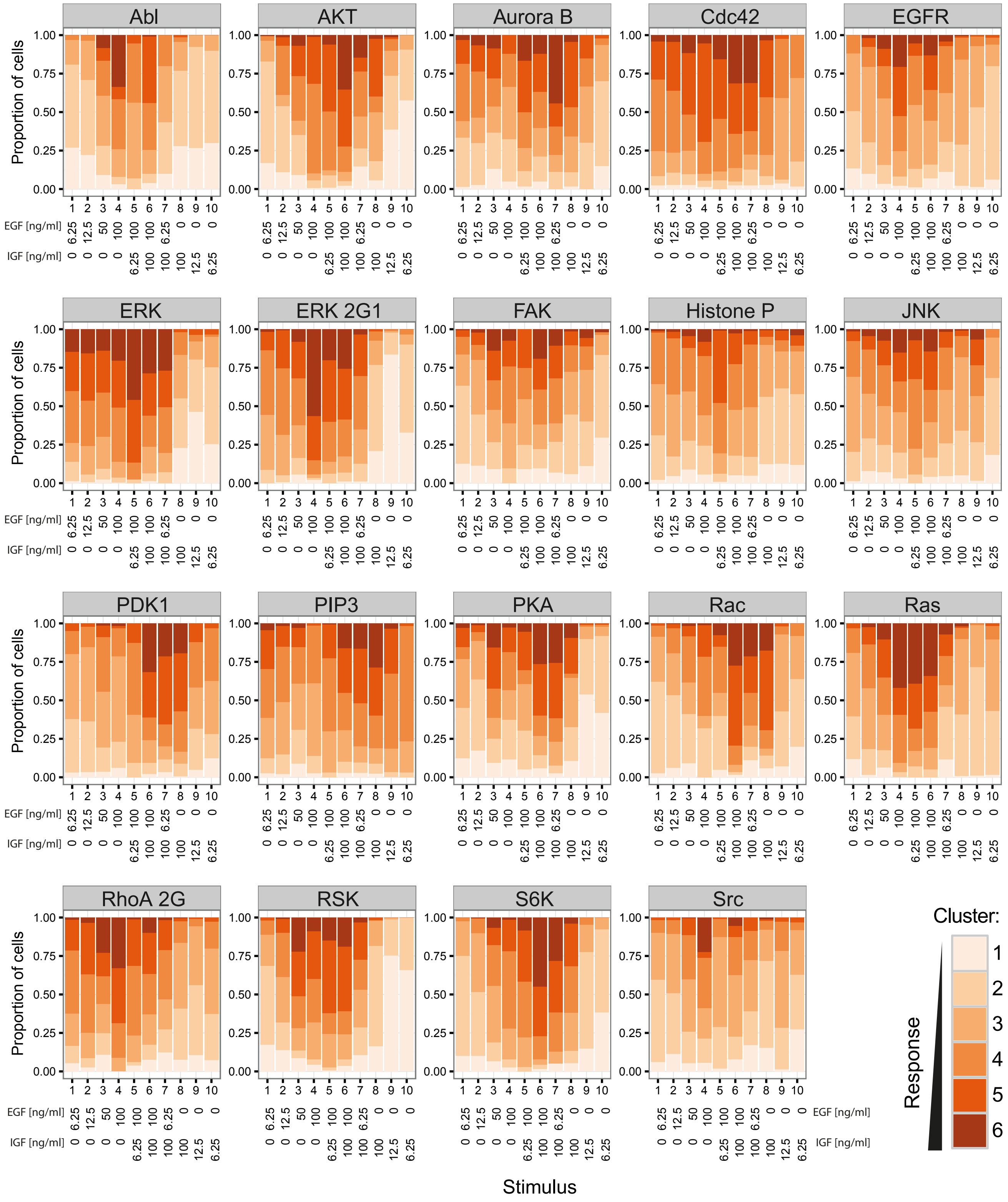

Supplementary Figure 12

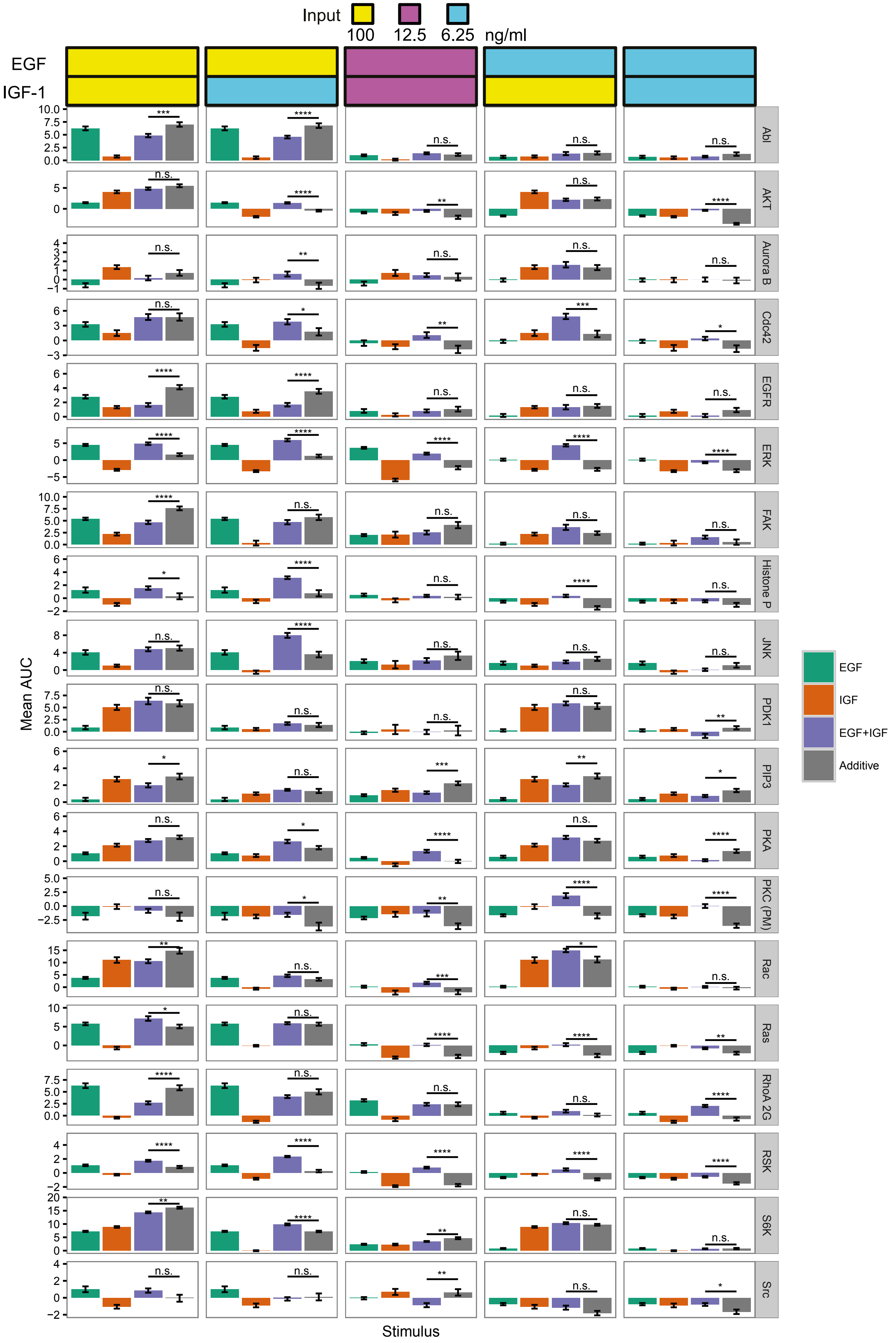

Supplementary Figure 13

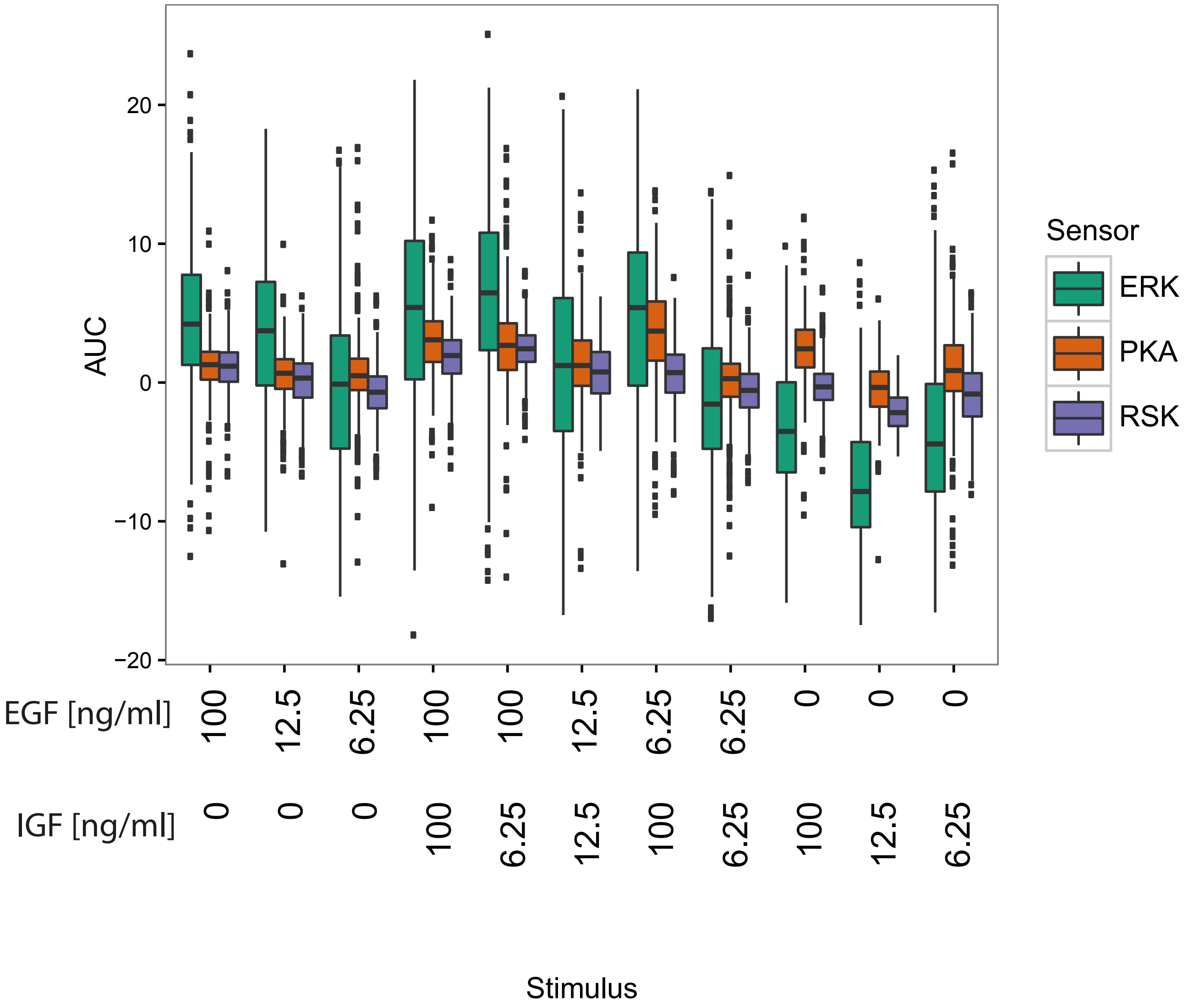

Supplementary Figure 14

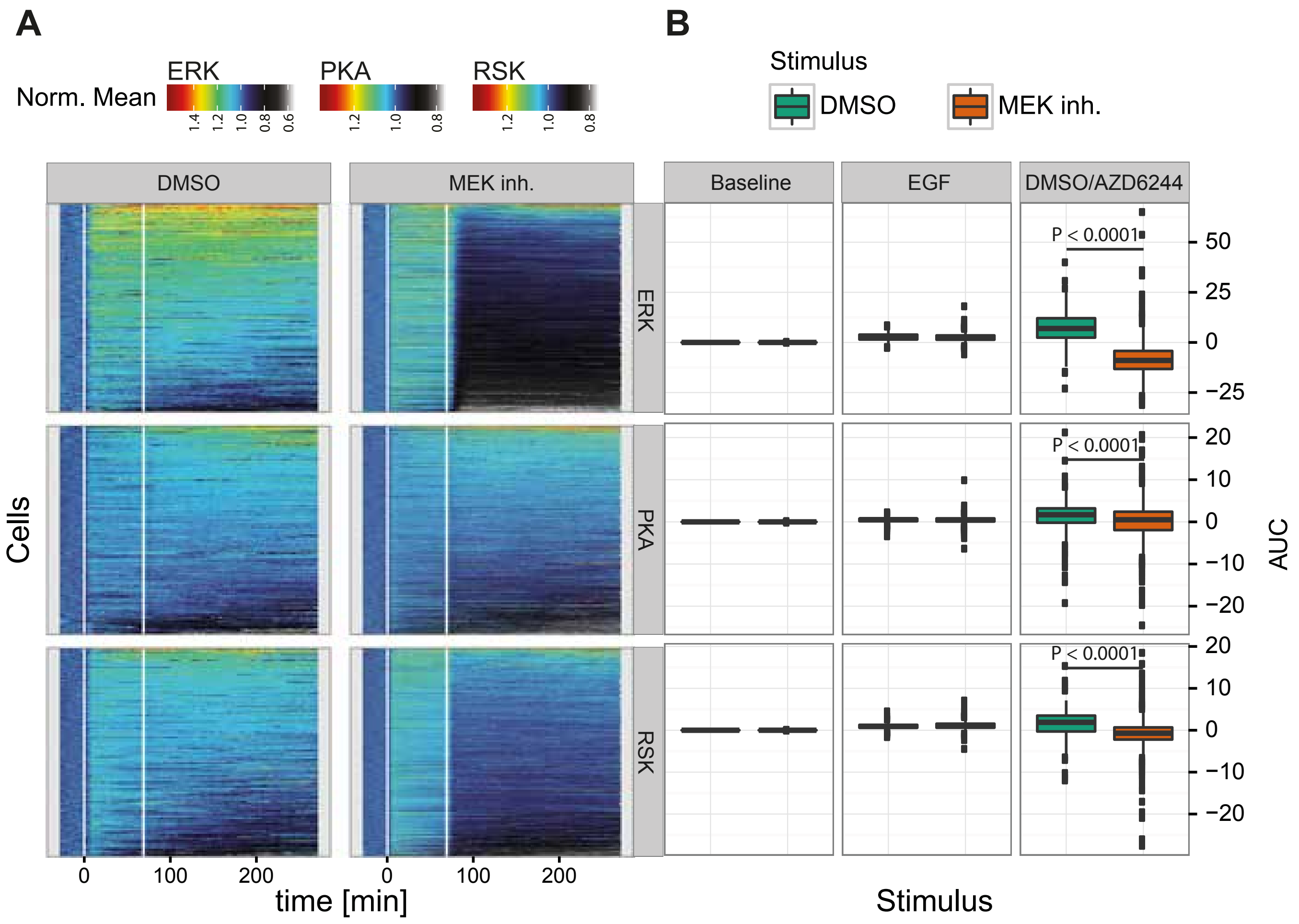

Supplemental Figures and Tables

Figure S1, related to Figure 1

Image analysis pipeline of an in-house developed macro.

Figure S2, related to Figure 1

Compatibility with variety of adherent cells and cross-contamination analysis.

(**A**) Images of cells expressing EKAREV (ERK) FRET biosensor. Scale bar, 200 µm. (**B**) Microarray of HeLa cells printed with plasmids expressing either CFP (blue) or GFP (yellow) in an alternating pattern. Bottom, higher magnification image of four spots from the array. (**C**) Quantification of cells expressing CFP and/or YFP on a spot containing CFP plasmid from figure S2B. Data represent mean of all spots ± SD. (180 spots, three independent experiments).

Figure S3, related to Figure 1

Activity of EGF signaling network in HeLa and H838 cells.

(**A**) FRET biosensors responded to EGF stimulation. HeLa cells were stimulated with 6.25 ng/mL, 12.5 ng/mL, 25 ng/mL or 100 ng/ml EGF at time 0. Data represent mean ± SEM ($n \geq 2$). (**B**) H838 cells were stimulated with 50 ng/mL of EGF at time 0. Data represent mean ± S.E. ($n = 4$). (**C**) FRET response of H838 cells expressing FRET biosensors after application of EGF (50ng/mL). Ras/ERK/RSK (left) and PDK1/Akt/S6K (right) pathways. Cells were stimulated at time 0. Data represent mean ± SEM ($n = 4$).

Figure S5, related to Figure 1

Heterogeneity in the response of HeLa cells expressing FRET biosensors upon EGF treatment.

(**A**) Coefficient of variation for each responding FRET biosensors from a single experiment was plotted against time. HeLa cells were stimulated with 100 ng/ml EGF at time 0. (**B**) Randomly selected single cell trajectories from the experiment depicted in Figure S5A.

Figure S6, related to Figure 1

The impact of the FRET biosensor expression level on cellular signaling.

(**A**) Monitoring FRET biosensor expression level and FRET ratio in untreated cells. HeLa cells were stimulated with imaging medium at time 0 to mimic adding of growth factor. FRET biosensor expression levels were approximated to the intensity of donor channel (CFP) before stimulation. Data represent mean ± *SEM* ($n \geq 3$) (**B**) Correlation between the maximum of normalized response and FRET biosensor expression levels. HeLa cells were stimulated with 100 ng/ml EGF ($n > 65$, depending on the FRET biosensor). FRET biosensor expression levels were approximated by averaging 10 frames of donor channel (CFP) before stimulation. r: Kendall's correlation coefficient. p: p-value.

Figure S7, related to Figure 2

Pharmacological perturbation of EGF signaling network activity by a MEK inhibitor (AZD6244).

(**A**) Strongly effected signaling molecules by AZD6244 and (**B**) signaling molecules showing moderate or no effect upon adding AZD6244. HeLa cells were stimulated with EGF (100 ng/mL) at time 0 and treated with DMSO or AZD6244 (5 µM) after 69 min. Data represent mean ± SEM ($n = 2$, DMSO, and $n=3$, AZD6244).

Figure S8 , related to Figure 2

Perturbation of EGF signaling by expression of constitutively active EGFR.

H838wt or H838 cells expressing EGFR with both activating L858R and resistant T790M mutations were stimulated with 50 ng/mL of EGF at time 0. Data represent mean ± *SEM* (n = 3).

Figure S9, related to Figure 3

Example of heterogeneous dynamics of protein activity under various conditions in HeLa cells.

Each line of the heatmaps represents a single cell. HeLa cells were treated with 12.5 ng/ml EGF, 100 ng/ml EGF, 100 ng/ml EGF + 100 ng/ml IGF-1, 100 ng/ml IGF-1 at time 0 (vertical white line) (100< n <450).

Figure S10, related to Figure 3

Cluster representative time series.

For each biosensor, normalized to untreated cells time series from all experiments were subjected to K-means clustering with squared euclidian distance. The representative cluster trajectories are color coded. Data represent mean ± *SEM.*

Figure S11, related to Figure 3

Distribution of representative FRET biosensors time series in response to various stimuli.

HeLa cells were treated with 1 – 6.25 ng/ml EGF, 2 – 12.5 ng/ml EGF, 3 – 50 ng/ml EGF, 4 – 100 ng/ml EGF, 5 – 100 ng/ml EGF + 6.25 ng/ml IGF-1, 6 – 100 ng/ml EGF + 100 ng/ml IGF-1, 7 – 6.25 ng/ml EGF + 100 ng/ml IGF-1,8 – 100 ng/ml IGF-1, 9 – 12.5 ng/ml IGF-1,10 – 6.25 ng/ml IGF-1. The clusters are color coded. n > 1296 cells for each biosensor.

Figure S12, related to Figure 4

Difference between calculated (or expected) and experimental additivity.

Area under the curve after stimulation is calculated for each single cell and depicted as mean ± *SEM*. We calculated the expected response (grey) by simple addition of the mean of the area under the curve of "EGF only" and "IGF-1 only" treatments. ****p < 0.0001, ***p < 0.001, **p < 0.01 and *p < 0.05, n.s. ≥ 0.05.

Figure S13, related to Figure 4

PKA activity is correlated with ERK and RSK activities in the presence of EGF.

Area under the curve of single cells plotted against various growth factor treatments. HeLa cells were treated with 6.25 ng/ml EGF, 12.5 ng/ml EGF, 50 ng/ml EGF, 100 ng/ml EGF, 100 ng/ml EGF + 6.25 ng/ml IGF-1, 100 ng/ml EGF + 100 ng/ml IGF-1, 6.25 ng/ml EGF + 100 ng/ml IGF-1, 100 ng/ml IGF-1, 12.5 ng/ml IGF-1, 6.25 ng/ml IGF-1. 100 < n < 542 cells for each biosensor. Horizontal lines indicate the median, boxes the 25th–75th percentile, vertical lines the total range, and (▪) the outliers for each population

Figure S14, related to Figure 2

Single cell analysis of the MEK inhibitor induced decrease in PKA, ERK and RSK activity.

(**A**) Single cell dynamics of PKA, RSK and ERK activity. Data come from figure S7. Each line of the heatmaps represents a single cell. 347 < n < 1000 cells for each biosensor. (**B**) Distribution of ERK, PKA and RSK activity in cells. Area under the curve was calculated for each single cell: before stimulation (baseline), between EGF and DMSO/AZD6244 stimulation and after DMSO/AZD6244. HeLa cells were stimulated with EGF (100 ng/mL) at time 0 and treated with DMSO or AZD6244 (5 μM) after 69 min. Horizontal lines indicate the median, boxes the 25th–75th percentile, vertical lines the total range, and (▪) the outliers for each population

Table S1, related to Figure 1
FRET biosensors used in this study

Target	Sensor Name	Domains	Named In this work	Ref
ERK	EKAREV	WW (phosphopeptide binding domain)-Substrate domain (PDVPRTPVDKAKLSFQFP)	ERK EV	(Komatsu et al., 2011)
ERK	EKAR2G1	WW (phosphopeptide binding domain) - Substrate domain (PDVPRTPVDKAKLSFQFP)	ERK2G1	(Fritz et al., 2013)
RhoA	RhoA2G	RhoA-binding domain (RBD) of rhotekin– RhoA	RhoA	(Fritz et al., 2013)
Ras	RaichuEV-Ras (PM)	H-Ras domain Raf RBD – Ras-binding domain of Raf 1	Ras	(Komatsu et al., 2011)
Rac1	RaichuEV-Rac1 (PM)	PAK CRIB domain - Cdc42/Rac-interactive binding Rac1<br>K-Ras CT domain – targeting to plasma membrane	Rac	(Komatsu et al., 2011)
RSK	Eevee-RSK	FHA1 domain (phosphopeptide binding Ser-1798 of TSC2) - Substrate domain (GQRKRLITSVDDFTE)<br>NES	RSK	(Komatsu et al., 2011)
S6K	Eevee-S6K	FHA1 domain (phosphopeptide binding<br>Thr-1135 of Rictor) - Substrate domain (NRRIRTLTEPDVDFN)<br>NES	S6K	(Komatsu et al., 2011)
Akt	Eevee-Akt	Akt PH domain - FHA1 domain (phosphopeptide binding) - Substrate domain (RKRDRLGTLGD)<br>Akt substrate<br>NES	Akt	(Komatsu et al., 2011)
PKC	Eevee-PKC	PKCβ C1 domain - FHA1 domain (phosphopeptide binding) - Substrate domain (KKKKKRFTFKDSFKL)<br>NES	PKC EV	(Komatsu et al., 2011)
JNK	JNKAR1EV	FHA1-EV- Substrate domain (DSVKTPEDEGNPLLEQLEKK)	JNK	(Komatsu et al., 2011)
EGFR/abl	PicchuEV	CrkII-SH2-SH3 domain-<br>217–225 residues of CrkII	EGFR	(Komatsu et al., 2011)

		(EPGPYAQPS)		
PIP3/PI(3,4)P2	InPAkt pmInPAKt (PM)	PH AKT -pseudoligand	PIP3	(Ananthanarayanan et al., 2005)
Src	Src biosensor	CFP-SH2 (Src)- Substrate (synthetic)	Src	(Ouyang et al., 2008)
Cdc42	Raichu-Cdc42 (PM)	PAK1-Cdc42 (EV linker was cloned in between PAK1 and Cdc42)	Cdc42	(Itoh et al., 2002)
PAK1	Pakabix (PM) Pakabi	human PAK1, Pakabix carries the C-terminal region of Ki-Ras4B	PAK (PM) PAK	(Parrini et al., 2009)
Calcineurin	CaNARI	domain of NFAT1	Calcineurin	(Newman and Zhang, 2008)
FAK	FAK biosensor	SH2(c-Srk) -substrate	FAK	(Seong et al., 2011)
cRaf	Prin-cRaf	cRaf	cRaf	(Terai and Matsuda, 2005)
bRaf	Prin-bRaf	bRaf	bRaf	(Terai and Matsuda, 2006)
Histone H3-K9 methylation	H3 K9 reporter	HP1 Chromodomain-histone H3	Histone K9 Me	(Lin et al., 2004)
Histone H3-K27 methylation	H3 K27 reporter	Polycomb (Pc) Chromodomain-histone H3	Histone K27 Me	(Lin et al., 2004)
Histone H3-S28 phosphorylation	H3 S28 reporter	14-3-3t – 615-644 residues of Cbl	Histone P	(Lin and Ting, 2004)
Cdk1	Cdk1 Sensor	Polo-Box Domain of Plk1-(GGT)5 linker-Cyclin B1 containing the Ser126 autophosphorylation site	Cdk1	(Gavet and Pines, 2010)
PDK1	PARE (PM)	PDK1	PDK1	(Gao et al., 2011)
Caspase-3	Caspase-3 reporter	DEVDR	Caspase-3	(Albeck et al., 2008)
Caspase-8/10	Caspase-8/10 reporter	IETD2x	Caspase-8/10	(Albeck et al., 2008)
PKA	AKAR3EV	FHA1 domain - PKA substrate (LRRATLVD) - NES	PKA	(Komatsu et al., 2011)
PKC	erCKAR (ER) pmCKAR (PM)	FHA2 (Rad53P)-Substrate(synthetic)	PKC (ER)	(Violin et al., 2003)
Rap1	Raichu-Rap1 (PM)	Rap1A-Raf	Rap1	(Mochizuki et al., 2001)
RalA	Raichu-RalA (PM)	RalA-RalBP1	RalA	(Takaya et al., 2004)
RhoA	RhoA	PKN -RhoA	RhoA	(Yoshizaki et al., 2003)
Ca2+	TN-XL	chicken skeletal muscle TnC	Ca2+ (TNXL)	(Mank et al., 2006)

CaMKII	CaMK2α	CaMK2α	Ca2+	(Piljic et al., 2011)
Ca2+	Twitch	TnC domain	Ca2+ (Twitch)	(Thestrup et al., 2014)
Pickles	Abl	CrkL	Abl	(Mizutani et al., 2010)
Aurora B	Aurora B (chromatin)<br>Aurora B (cytosol)<br>Aurora B (centromer)	FHA2-substrate(Kif2 57-70)	Aurora B H2B<br><br>Aurora B<br><br>Aurora B centro	(Fuller et al., 2008)
Control	C32V		control	(Koushik et al., 2006)
Control 2	DEVG	DEVDR mutated to DEVG	DEVG	(Albeck et al., 2008)

Albeck, J.G., Burke, J.M., Spencer, S.L., Lauffenburger, D.A., and Sorger, P.K. (2008). Modeling a snap-action, variable-delay switch controlling extrinsic cell death. PLoS biology *6*, 2831-2852.

Ananthanarayanan, B., Ni, Q., and Zhang, J. (2005). Signal propagation from membrane messengers to nuclear effectors revealed by reporters of phosphoinositide dynamics and Akt activity. Proceedings of the National Academy of Sciences of the United States of America *102*, 15081-15086.

Fritz, R.D., Letzelter, M., Reimann, A., Martin, K., Fusco, L., Ritsma, L., Ponsioen, B., Fluri, E., Schulte-Merker, S., van Rheenen, J., et al. (2013). A versatile toolkit to produce sensitive FRET biosensors to visualize signaling in time and space. Science signaling *6*, rs12.

Fuller, B.G., Lampson, M.A., Foley, E.A., Rosasco-Nitcher, S., Le, K.V., Tobelmann, P., Brautigan, D.L., Stukenberg, P.T., and Kapoor, T.M. (2008). Midzone activation of aurora B in anaphase produces an intracellular phosphorylation gradient. Nature *453*, 1132-1136.

Gao, X., Lowry, P.R., Zhou, X., Depry, C., Wei, Z., Wong, G.W., and Zhang, J. (2011). PI3K/Akt signaling requires spatial compartmentalization in plasma membrane microdomains. Proceedings of the National Academy of Sciences of the United States of America *108*, 14509-14514.

Gavet, O., and Pines, J. (2010). Progressive activation of CyclinB1-Cdk1 coordinates entry to mitosis. Developmental cell *18*, 533-543.

Itoh, R.E., Kurokawa, K., Ohba, Y., Yoshizaki, H., Mochizuki, N., and Matsuda, M. (2002). Activation of rac and cdc42 video imaged by fluorescent resonance energy transfer-based single-molecule probes in the membrane of living cells. Molecular and cellular biology *22*, 6582-6591.

Komatsu, N., Aoki, K., Yamada, M., Yukinaga, H., Fujita, Y., Kamioka, Y., and Matsuda, M. (2011). Development of an optimized backbone of FRET biosensors for kinases and GTPases. Molecular biology of the cell *22*, 4647-4656.

Koushik, S.V., Chen, H., Thaler, C., Puhl, H.L., 3rd, and Vogel, S.S. (2006). Cerulean, Venus, and VenusY67C FRET reference standards. Biophysical journal *91*, L99-L101.

Lin, C.W., Jao, C.Y., and Ting, A.Y. (2004). Genetically encoded fluorescent reporters of histone methylation in living cells. Journal of the American Chemical Society *126*, 5982-5983.

Lin, C.W., and Ting, A.Y. (2004). A genetically encoded fluorescent reporter of histone phosphorylation in living cells. Angewandte Chemie *43*, 2940-2943.

Mank, M., Reiff, D.F., Heim, N., Friedrich, M.W., Borst, A., and Griesbeck, O. (2006). A FRET-based calcium biosensor with fast signal kinetics and high fluorescence change. Biophysical journal *90*, 1790-1796.
Mizutani, T., Kondo, T., Darmanin, S., Tsuda, M., Tanaka, S., Tobiume, M., Asaka, M., and Ohba, Y. (2010). A novel FRET-based biosensor for the measurement of BCR-ABL activity and its response to drugs in living cells. Clinical cancer research : an official journal of the American Association for Cancer Research *16*, 3964-3975.
Mochizuki, N., Yamashita, S., Kurokawa, K., Ohba, Y., Nagai, T., Miyawaki, A., and Matsuda, M. (2001). Spatio-temporal images of growth-factor-induced activation of Ras and Rap1. Nature *411*, 1065-1068.
Newman, R.H., and Zhang, J. (2008). Visualization of phosphatase activity in living cells with a FRET-based calcineurin activity sensor. Molecular bioSystems *4*, 496-501.
Ouyang, M., Sun, J., Chien, S., and Wang, Y. (2008). Determination of hierarchical relationship of Src and Rac at subcellular locations with FRET biosensors. Proceedings of the National Academy of Sciences of the United States of America *105*, 14353-14358.
Parrini, M.C., Camonis, J., Matsuda, M., and de Gunzburg, J. (2009). Dissecting activation of the PAK1 kinase at protrusions in living cells. The Journal of biological chemistry *284*, 24133-24143.
Piljic, A., de Diego, I., Wilmanns, M., and Schultz, C. (2011). Rapid development of genetically encoded FRET reporters. ACS chemical biology *6*, 685-691.
Seong, J., Ouyang, M., Kim, T., Sun, J., Wen, P.C., Lu, S., Zhuo, Y., Llewellyn, N.M., Schlaepfer, D.D., Guan, J.L., et al. (2011). Detection of focal adhesion kinase activation at membrane microdomains by fluorescence resonance energy transfer. Nature communications *2*, 406.
Takaya, A., Ohba, Y., Kurokawa, K., and Matsuda, M. (2004). RalA activation at nascent lamellipodia of epidermal growth factor-stimulated Cos7 cells and migrating Madin-Darby canine kidney cells. Molecular biology of the cell *15*, 2549-2557.
Terai, K., and Matsuda, M. (2005). Ras binding opens c-Raf to expose the docking site for mitogen-activated protein kinase kinase. EMBO reports *6*, 251-255.
Terai, K., and Matsuda, M. (2006). The amino-terminal B-Raf-specific region mediates calcium-dependent homo- and hetero-dimerization of Raf. The EMBO journal *25*, 3556-3564.
Thestrup, T., Litzlbauer, J., Bartholomaus, I., Mues, M., Russo, L., Dana, H., Kovalchuk, Y., Liang, Y., Kalamakis, G., Laukat, Y., et al. (2014). Optimized ratiometric calcium sensors for functional in vivo imaging of neurons and T lymphocytes. Nat Methods *11*, 175-182.
Violin, J.D., Zhang, J., Tsien, R.Y., and Newton, A.C. (2003). A genetically encoded fluorescent reporter reveals oscillatory phosphorylation by protein kinase C. The Journal of cell biology *161*, 899-909.
Yoshizaki, H., Ohba, Y., Kurokawa, K., Itoh, R.E., Nakamura, T., Mochizuki, N., Nagashima, K., and Matsuda, M. (2003). Activity of Rho-family GTPases during cell division as visualized with FRET-based probes. The Journal of cell biology *162*, 223-232.
